# Controlling drug-resistant bacteria in Arabian horses: bacteriophage cocktails for treating wound infections

**DOI:** 10.3389/fvets.2025.1609955

**Published:** 2025-10-15

**Authors:** Esraa Khalid, Yasmine H. Tartor, Ahmed M. Ammar, Rewan Abdelaziz, Yasser Mahmmod, Adel Abdelkhalek

**Affiliations:** ^1^Department of Microbiology, Faculty of Veterinary Medicine, Zagazig University, Zagazig, Egypt; ^2^Department of Microbiology, Faculty of Science, Ain Shams University, Cairo, Egypt; ^3^Department of Veterinary Clinical Sciences, College of Veterinary Medicine, Long Island University, Brookville, NY, United States; ^4^Department of Food Safety, Hygiene and Technology, Faculty of Veterinary Medicine, Badr University in Cairo (BUC), Badr, Egypt

**Keywords:** Arabian horses, one health, antimicrobial resistance, bacteriophage, biosecurity, wound, *Acinetobacter baumannii*, methicillin-resistant *Staphylococcus aureus*

## Abstract

Antimicrobial resistance is a major global health issue requiring a coordinated response. This study investigated for the first time the prevalence, antimicrobial resistance phenotypes of bacteria causing infections in Arabian horses, and the potential of bacteriophage therapy for wound treatment. One hundred clinical samples from infected Arabian horses, presenting respiratory disorders, diarrhea, abortion, wound, and ocular infection, were examined using direct sample multiplex PCR and phenotypic methods. Antimicrobial susceptibility testing of the recovered isolates was performed using panels of 37 antibiotics and broth microdilution method. Bacteriophages were isolated from horse manure. A bacteriophage cocktail was used for treating infected wounds in Arabian horses. *Streptococcus equi* was the most predominant pathogen isolated from respiratory infections (17/29, 58.6%), followed by *Klebsiella pneumoniae* and *Pseudomonas aeruginosa* (9/29, 31.03%, each), and *Escherichia coli* (7/29, 24.13%). *Staphylococcus aureus* and *Corynebacterium ovis* biovar *equi* were the most frequently isolated bacteria from pyogenic infections. All isolated bacteria showed resistance to multiple antibiotics. *Streptococcus* spp. exhibited extensive drug resistance (XDR) with complete resistance to amoxicillin-clavulanic acid, amikacin, kanamycin, streptomycin, and cefotaxime. All *Staphylococcus* spp. displayed multidrug resistance (MDR) phenotype. *Staphylococci* isolates were highly resistant to fusidic acid, *β*-lactams, and tetracyclines. Amoxicillin-clavulanic acid, fosfomycin, and cephalosporines were ineffective against *Enterobacteriaceae* isolates. Ticarcillin, clavulanic acid, and colistin were ineffective against *P. aeruginosa* and *Acinetobacter baumannii*. Pan-drug-resistant (PDR) *P. aeruginosa* isolate was detected in the infected wound. Two lytic bacteriophages (vB_Pae_LP125 and vB_Pae_LS225) from the *Podoviridea* and *Siphoviridea* families were isolated from the horse manure. Both phages were stable across various temperatures and pH levels. *In vitro* tests showed significant lytic activity against a wide range of bacterial strains. The DNA genomes of all phages displayed distinctive restriction fragment length polymorphism. A bacteriophage cocktail (vB_Pae_LP125 and vB_Pae_LS225), when combined with gentamicin, improved wound healing in infected horses. There were significant differences (*p* < 0.05) in the wound closure % among the gentamicin group and phage cocktaoil+gentamicin groups on days 3, 5, 7, 10, and 14. This study highlights the widespread antibiotic resistance in bacteria infecting Arabian horses and posing significant challenges to equine infection management. Bacteriophage therapy shows promise as a potential treatment for wound infections.

## 1 Introduction

Bacterial infections are the main cause of economic loss in horses (1). They greatly affect the welfare of horses and pose critical challenges in veterinary medicine. The European Union launched its ‘One Health’ strategy in 2017 to fight antibiotic resistance in animal and human health and to reduce the transmission of zoonotic diseases (2). Among the various bacterial infections requiring heightened attention in treatment, respiratory tract infections represent the most prevalent source of significant bacterial isolates, followed by wound infections (3). Horses commonly incur wounds because of their tendency to fight and environmental factors (4). Equine ocular disorders are a major global medical concern, requiring prolonged, costly treatment and potentially decreasing the commercial value of affected horses (5). Additionally, foal diarrhea remains a severe global problem, serving as the leading cause of mortality among young foals (6). Approximately 80% of foals suffer from diarrhea at some stage in their lives, which can cause serious health complications such as dehydration, nutrient loss, and even death if left untreated (7). Furthermore, endometritis is a primary cause of reduced fertility in mares, which substantially affects the equine breeding industry by causing infertility and early embryonic loss (4). Abortion rates in horse pregnancies range from 10 to 15%, which is attributed to various infectious and non-infectious factors (8).

The rapid rise in antibiotic-resistant pathogens presents major global health and economic issues, worsened by the improper use of antibiotics in human and veterinary medicine. This has created significant challenges in treating infections caused by multidrug-resistant (MDR) pathogens, often leading to increased morbidity and mortality (9). The severity of this problem has prompted the classification for MDR bacteria. Worrisome are extensively drug-resistant (XDR) strains, susceptible to only two antimicrobial classes, and pan-drug-resistant (PDR) bacteria that are resistant to all known antimicrobial categories (10). These trends underscore the critical and urgent nature of the antimicrobial resistance issue (2).

Given the rise in antimicrobial resistance and its potential negative effects on equines, it is crucial to detect infectious diseases early, choose appropriate first-line antimicrobials, and promptly discontinue treatment when suitable (1). International approaches plan and collaborative multisectoral approach are essential to address and curb the spread of AMR. The key to these efforts is the discovery of new antimicrobial agents and the application of advanced biotechnological methods to develop alternative antimicrobial strategies (1, 9). While extensive research has been conducted on antimicrobial resistant bacteria causing infections in equines thoroughbreds, there is limited focus on the Arabian breed (6, 11, 12).

Researchers have investigated unconventional treatments, among these is bacteriophages, their broad diversity and selectivity make them promising therapeutic candidates for the treatment and prevention of MDR bacteria (9). They have many advantages over traditional antibiotics in treating infectious diseases. Specifically, potency, host-limited immune response, and their capability for self-proliferation and bioengineering. In addition, they are non-toxic and harmless to normal flora. For host bacterial species, phage specificity is a significant benefit. These characteristics have led to their consideration as potential antibiotic alternatives (2, 9). Therefore, they can be used to treat bacterial infections either alone or in combination with antibiotics (13).

Phage therapy has been explored in equine medicine, mainly targeting superficial skin and ocular infections (14, 15). Despite their promising results, these studies have encountered significant limitations the narrow host range of phages, allowing non-target bacteria to persist. This work represents one of the earliest attempts to apply bacteriophage therapy in the treatment of bacterial infections specifically in Arabian horses. The present study represents one of the earliest attempts to implement bacteriophage therapy for the treatment of bacterial infections in Arabian horses. To address the limitations of previous research, this work utilizes broad-spectrum phage cocktails and investigates their combined use with antibiotics to improve efficacy and reduce antimicrobial resistance.

This study aimed to (i) investigate the prevalence of antimicrobial-resistant bacteria causing infections in Arabian horses, (ii) isolate and characterize bacteriophages with strong lytic ability, and (iii) explore the applications of bacteriophage cocktail for treating the infected wounds in Arabian horses.

## 2 Materials and methods

### 2.1 Study population and clinical samples

One hundred samples were collected from infected Arabian horses (aged 10 days −23 years) from different stations in Egypt and from private cases in Sharkia and Ismailia governorates over one-year, from August 2022 to August 2023. Swabs were collected from respiratory infections, wounds, and diarrhea. Uterine fluid was obtained from cases suffering from abortion and endometritis (Supplementary Table S1). The samples were collected before initiating antibiotics treatment. Swabs were transported to the laboratory in an ice container (0–4 °C) and transported to the laboratory within an hour of collection for immediate bacteriological examination. A specimen from each case was incubated overnight in Brain Heart Infusion (BHI) broth (Oxoid, United States).

### 2.2 Isolation and identification of the causative agents

Respiratory samples were plated on BHI agar, MacConkey agar, and Edward’s medium. Swabs from infected wound cases and pus from pyogenic infections (abscesses and open wounds), as illustrated in Supplementary Figures S1A–D, were cultured on mannitol salt agar (MSA), MacConkey agar, and BHI agar. Rectal swabs from diarrheal cases were enriched in Rappaport Vassiliadis broth at 41 °C for 24 h, followed by plating on xylose-lysine-deoxycholate (XLD) agar and incubation at 37 °C for 24 h. All media were sourced from Oxoid, United States.

The isolates were examined microscopically using Gram staining, and bacterial identification was performed using standard biochemical tests; for example, catalase and coagulase tests were performed to identify *Staphylococci* (16). Presumptive Gram-negative bacteria were identified through different biochemical tests such as urease, citrate, oxidase, lysine decarboxylase, and triple sugar iron tests (Oxoid, United Kingdom) (17).

### 2.3 DNA extraction and PCR assays

#### 2.3.1 Preparation of DNA from samples

For direct-sample PCR, 54 clinical swabs, representing different infections and localities, were incubated in 4 mL BHI broth at 37 °C overnight. BHI broth (1 mL) was centrifuged at 21,000 xg for 10 min (18). The extraction steps were performed according to the QIAamp DNA Mini Kit guidelines (Catalog no. 51304, QIAGEN®, Hilden, Germany) according to the manufacturer’s instructions. DNA concentration and purity were assessed using Thermo Scientific™ NanoDrop 2000/2000c.

#### 2.3.2 PCR amplification and cycling conditions

Multiplex PCR was performed using a thermal cycler (Biometra, Germany) in a 50 μL reaction mixture containing 25 μL of Emerald Amp GT PCR MasterMix (Code No. RR310A Takara, United States) (2x premix), 1 μL of each forward and reverse primers (20 pmol; Biobasic, Canada), 15 μL of PCR-grade water, and 6 μL of DNA template. Each run included positive and negative controls. The target genes and primer sequences used in this study are shown in Supplementary Table S2. Supplementary Table S3 summarize cycling conditions for multiplex PCR targeting multiple species including *E. coli*, *S. aureus*, *Corynebacteria* spp., *A. baumannii*, *Salmonella* spp., *S. pyogenes*, *S. equi*, *Pseudomonas* spp., and *K. pneumoniae*.

Positive samples for genus *Corynebacterium* were amplified using uniplex PCR targeting the *narG* gene for *Corynebacterium ovis* biovar *equi* (*C. ovis* biovar *equi*) (19). The conditions of thermal cycler were mentioned in Supplementary Table S4. Positive samples for *Pseudomonas* spp. were amplified using primer targeting *P. aeruginosa* 16S rRNA gene (20) (Supplementary Table S4).

Coagulase-negative *Staphylococci* (CoNS) were amplified using a specific primer for the 16S rRNA gene (21) (Supplementary Table S4). Additionally, fecal swab samples were amplified using species-specific primers targeting *invA* (22) and *ureR* genes (23) of *Salmonella enterica* spp. and *P. mirabilis*, respectively (Supplementary Table S4).

*Klebsiella pneumoniae* ATCC 10031, *C. pseudotuberculosis* ATCC 19410 T, *Streptococcus Equi subsp. zooepidemicus* ATCC 43079, *S. equi* ATCC 33398, *A. baumanni* ATCC 19606, *P. mirabilis* strain HI4320, *S. aureus* ATCC 33591 reference strains were included in each run as positive controls.

Electrophoresis of the PCR products was performed on a 1.5% agarose gel (Applichem GmbH, Darmstadt, Germany), using GelPilot 100 bp Plus Ladder (cat. no. 239045, QIAGEN, United States) and the gel was stained with 0.5 μg mL ethidium bromide. The bands were photographed, their sizes were determined using the Alpha Innotech gel documentation system (Biometra GmbH, Göttingen, Germany).

### 2.4 Antimicrobial susceptibility testing

Antimicrobial susceptibility of the recovered isolates was performed using the disk diffusion method according to Clinical and Laboratory Standards Institute (CLSI, 2024) guidelines (24) and interpretative criteria. A suspension of the tested isolate was prepared by adjusting the turbidity with 0.5 McFarland solution and then smeared on the surface of Muller-Hinton agar (MHA, Oxoid, Cambridge, United Kingdom), and the antibiotic disks (Oxoid, UK) were placed (Supplementary Tables S5A–E). Antibiotics to which susceptibilities of isolates were recorded to determine MDR, XDR, and PDR phenotypes were selected according to Magiorakos et al. (10) and Nocera et al. (25). The plates were incubated at 37 °C for 24 h, after which the inhibition zones diameters were measured and interpreted as sensitive (S) or intermediate (I) and resistant (R) according to CLSI documents (24) and if there is lacking information the guidelines from the European Committee on antimicrobial susceptibility testing (EUCAST, 2021) (26) were used (Supplementary Tables S5A–E). Quality control was assessed using the following strains *E. coli* ATCC 25922, *P. aeruginosa* ATCC 27853, *K. pneumoniae* ATCCBAA-1705, *S. aureus* ATCC 25923, *S. pyogenes* ATCC 12344, *S. equi* ATCC 33398, *A. baumannii* ATCC 17978, and *P. mirabilis* ATCC 29245.

The multiple antibiotic resistance (MAR) index for each isolate was calculated by dividing the number of antimicrobial agents to which an isolate exhibited resistance by the total number of antibiotics tested (27).

#### 2.4.1 Resazurin assay and determination of minimum inhibitory concentration (MIC) of antibiotics

The isolates were also examined using the broth microdilution method to determine the minimum inhibitory concentrations (MICs) of VA (Sigma-Aldrich, United States), TGC (Sigma-Aldrich, USA), and CT (Sigma-Aldrich, Seelze, Germany) according to CLSI and EUCAST guidelines (24, 26). Briefly, 100 μL Muller-Hinton broth (MHB, Oxoid, UK) was added to each well of a sterile 96-well plate, and serial double-fold dilutions of each antibiotic were performed in 10 wells in each vertical row. The concentration ranges used were as follows: CT, 0.125–64 μg/mL; VA 0.0625–64 μg/mL; TGC, 0.0625–64 μg/mL. Each bacterium for testing was prepared from MHB growth adjusted to a 0.5 McFarland tube. The suspension was then diluted 1:10 in MHB until a dilution of 1.5×10^6^ CFU/ml then 100 μL was added to all wells. The negative control well contained medium only, and the positive control well contained inoculum and medium. The plates were wrapped, coated in plastic bags, and incubated overnight at 37 °C. Resazurin indicator solution was prepared by dissolving 270 mg tablet (Sigma Aldrich, Germany) in 40 mL of sterile distilled water with concentration 6.75 mg/mL (0.675% w/v), using a vortex mixer to ensure complete dissolution, and kept in a brown bottle to prevent exposure to light at 4 °C for a maximum of 2 weeks from preparation. After the final visual reading, 10 μL (0.067 mg/mL) of resazurin indicator was added to each well, and the plate was further incubated for 2–4 h to observe the color change. Any color changes from blue to pink or colorless indicated bacterial growth. The lowest concentration before the color change was recorded as the MIC. The plate was rejected if the positive control wells remained unchanged (28).

*Staphylococcus aureus* isolates with (MIC of ≤2 μg/mL) is vancomycin-susceptible *S. aureus* (VSSA), *S. aureus* with (MIC of 4–8 μg/mL) is vancomycin intermediate *S. aureus* (VISA), and *S. aureus* with (MIC of ≥ 16 μg/mL) is vancomycin-resistant *S. aureus* (VRSA) (24).

As there are no CLSI breakpoints for TGC, the FDA breakpoints (29) were considered. For *S. aureus*: MIC ≤ 0.5 μg/mL is susceptible and more than that is considered resistant, while for *Enterobacteriaceae* spp. MIC ≤ 2 μg/mL is susceptible, 4 μg/mL is intermediate, and MIC ≥8 μg/mL is resistant. While colistin was considered sensitive at MIC ≤ 2 μg/mL and resistant at MIC ≥ 4 μg/mL (24).

### 2.5 Bacteriophage isolation

Manure samples from horses were collected, and bacteriophages were isolated using the double agar plate method (30). The samples were centrifuged at 21,840 × g for 10 min, and the supernatant was filtered through 0.45 μm syringe-driven filters. To inactivate residual bacterial cells and other microbial contaminants prior to phage enrichment, 50 mL of the filtrate was mixed with 0.5 mL of chloroform (final concentration ~1% v/v) and incubated for 20 min at room temperature, to preserve the infectivity of most tailed dsDNA phages while reducing bacterial contamination. Subsequently, 20 mL of 2 × Luria–Bertani broth (LB, Oxoid, Cambridge, United Kingdom) and 5 mL of the bacterial culture were added, and the mixture was incubated at 37 °C for 24 h. Broth cultures after 24 h were pelleted by centrifugation at 7600 × g at 4 °C for 20 min, and they were then filtered using 0.45 μm syringe-driven filters. The filtrate was then examined for lytic phage using the Adams double-layer agar method (31). The filtered samples were collected in sterile containers and stored at 4 °C (32).

### 2.6 Plaque assay and determination of bacteriophage titre

Plaque assays were used to determine phage titers. Briefly, serial dilutions of the phage suspension (10–1 to 10–6) were mixed with host bacteria and overlaid with semi-solid nutrient agar (Oxoid, Cambridge, United Kingdom) on nutrient agar plates. After incubation at 37 °C for 10 h, plaques were counted and PFU/mL was calculated using the standard formula (33).

### 2.7 Determination of phage host range

The host range of the two bacteriophages was assessed using the spot-test against the antimicrobial-resistant Gram-positive and Gram-negative isolates. Briefly, 100 μL of an overnight bacterial culture was spread onto NA plates and incubated for 3 h at 30 °C to allow partial growth. Then, 10 μL of each phage lysate was spotted onto the surface. Plates were incubated overnight at 30 °C, air-dried, and examined for lysis zones (34). To improve visualization and accommodate isolates with slower growth rates, a modified double-layer agar technique was also employed. Specifically, 100 μL of fresh broth culture was added to 50 mL of nutrient broth and incubated for 3 h at 37 °C. Then, 100 μL of this culture was mixed with 3 mL of molten top agar and overlaid onto nutrient agar plates. Ten microlitres of bacteriophage lysate were spotted onto the surface, and plates were incubated overnight. Lytic activity was recorded based on the appearance of clear zones.

### 2.8 Characterization of phage

#### 2.8.1 Thermal, ultraviolet light, and pH stability

The phages were subjected to heat and pH stability tests using a modified methodology from a previous study (35). The phage suspensions were incubated in a water bath in sterile Eppendorf for 10 min at various temperatures (37, 45, 55, 65, 70, and 80 °C). The plaque assay was used to determine the phage survival rate.

Phage stability at various pH values (1, 3, 5, 7, 9, 11, and) was determined using 1 mol/L HCl or 1 mol/L NaOH (36). One milliliter of each phage suspension was mixed with 9 mL of nutrient broth media at a specific pH value, incubated at 37 °C for 24 h and then tested using a plaque assay.

To assess the environmental stability of the isolated phages, ultraviolet (UV) sensitivity was evaluated. This experiment aimed to determine the resilience of phage infectivity under UV exposure, which is relevant for potential applications. Phage suspensions were exposed to UV light using a Cosmolux UVA lamp (Model A1-11-40 W PREHEAT-BIPIN, Germany), positioned at a fixed distance of 15 cm. The lamp emits UVA radiation in the range of 315–400 nm with a nominal power of 40 W. Phage samples were exposed for 0, 20, 40, 60, 80, 100, and 120 min in uncovered small plates. At each time point, aliquots were withdrawn and subjected to plaque assays to quantify residual infectivity. This approach allowed us to monitor the decline in phage viability over time and infer their sensitivity to UV-induced damage.

#### 2.8.2 Morphological characteristics of phages

The phage titer (approximately 10^10^ PFU/ml) was diluted tenfold in 1X phosphate-buffered saline (PBS) in 1 L of dsH_2_O (pH 7). A phage suspension was applied to the film surface (formal carbon) using 200 mesh copper grids. Uranyl acetate 2% (Micro Technologies, Myanmar, United States) was added for negative staining. The grids were air-dried after passing through a filter paper and examined using a transmission electron microscope (JEOL M-1400, Ontario, Canada) at 100 KV to determine the morphology and size of the phage.

The phage was classified using morphological criteria following the International Committee on Taxonomy of Viruses (ICTV) recommendations (37).

### 2.9 Phage adsorption assay

Adsorption of the phage onto the host cell was determined as previously described (38). The phage was added to an exponentially growing bacterial culture and incubated at room temperature without stirring. At several time points (1, 3, 5, 7, 9, 12, 15, 18, 21, 24, 27, and 30 min), 100 μL aliquots of the phage-bacterial combination were obtained and immediately centrifuged at 16,000 x for 30 s. Phage titers in the supernatant were then calculated by counting the plaques on overlay agar plates.

### 2.10 One-step growth technique

According to a previously described method (39), the bacteriophage latent time and burst size were assessed using a one-step growth technique. Fifty milliliters of broth culture were incubated until OD = 0.4 (3.0 × 10^8^ CFU/mL). The bacterial cells were then pelletized. The pelleted cells were resuspended in LB broth. To adsorb bacterial cells, approximately 0.5 mL of phage filtrate (3 × 10^8^ phage/mL) was added. The mixture (MOI = 1, calculated as the ratio of 3 × 10^8^ PFU/mL phage to 3 × 10^8^ CFU/mL bacterial cells) was centrifuged at 14,000 rpm for 30 min to remove free unabsorbed bacteriophages. The resulting pellet was resuspended in 100 mL LB broth and incubated at room temperature with continuous shaking at 120 rpm. Samples were collected from the culture flask every 3 min, and the phage titer was determined using the plaque assay.

### 2.11 Phage DNA extraction and restriction fragment length polymorphisms (RFLPs) analysis

DNA was extracted from each purified high-titer phage stock suspension using the DNeasy Blood and Tissue Kit (Qiagen, Hilden, Germany), following the manufacturer’s instructions (40). Phage genomic DNA was digested with different restriction enzymes namely, *hinf*I (Cat. No. FD0804), *hind* III (Cat. No. FD0504), and *hae*III (Cat. No. FD0154) according to the instructions of the manufacturer (Thermo FastDigest®). This process was performed at 37 °C for 15 min using a thermoshaker (Biometra, Germany). The 5 μL of restriction fragments was loaded onto a 1.0% agarose gel stained with ethidium bromide and visualized on a FluorChem gel documentation system (Alpha Innotech, San Leandro, CA, United States).

### 2.12 Application of phage for treating infected wound

The study population consisted of 12 horses presenting with purulent discharge from wound lesions at various body sites. Before the initiation of bacteriophage therapy, bacteriological examination was done on the swab samples obtained from the lesions. Horses were excluded from the study if they exhibited no clinical signs of infection and if no bacterial growth was detected. Upon admission for treatment, the 12 horses were assigned to three groups. Four horses in the first group received gentamicin (either topically or by intravenous injection at 6.6 mg/kg IV q24h (41) based on wound characteristics). Four horses in the second group received topical gentamicin combined with 4 mL bacteriophage cocktail (vB_Pae_LS225 and vB_Pae_LP125 which have wide host ranges). The final group of four horses received intravenous gentamicin alongside the topical application of the same bacteriophage cocktail.

Bacteriophage cocktail preparation (with approximately 2.2 ×10^9^ pfu/ml for vB_Pae_LP125 and 1.5×10^8^ pfu/ml for vB_Pae_LS225) was applied directly to the infected site in a 4 mL volume once every 24 h for 14 consecutive days.

A blind assessment was done for examination of wound closure on days 3, 5, 7, 10, 12, and 14- post treatment. Bacterial clearance was examined by culturing swab samples from wound lesions. The horses were examined also on day 21 for recurrence evaluation. The wound closure percentage was calculated using the following formula: Wound closure % = [V0 − Vt / V0] × 100.

Where V0 represents wound size at time zero and Vt is the wound size at time t (days).

The study protocol was approved by the Zagazig University Institutional Animal Care and Use Committee (approval no. ZU-IACUC/2/F/172/2024). Written informed consent was obtained from the owners for the participation of their animals in this study.

### 2.13 Data analysis

All experimental procedures were performed in triplicate in independent experiments. The results are expressed as the mean ± standard error of the mean (SEM). The normality and homogeneity of variance among the data were determined using Shapiro–Wilk’s and Levene’s tests, respectively. Data were analyzed using SPSS version 26 (IBM Corp, Armonk, NY, United States). The chi-square test was used to study the variations in the prevalence of different bacterial species from different origins, and to assess the differences in the antimicrobial resistance patterns of the recovered isolates from various sources. One-way ANOVA and Tukey’s post-hoc tests were used for analysis results of phage stability studies (thermal, pH, UV, one-step growth, and adsorption curves). Additionally, the independent sample *t*-test was performed to detect if there is a significant difference between the effect of two phages (two independent normal distributed groups) at each point (thermal, pH, UV, one-step growth, and adsorption curves). Analysis of phage treatment results were done using ANOVA and Tukey’s *post hoc* test. The significance level was set at *p* < 0.05. All graphs were generated using GraphPad Prism software version 8 (San Diego, CA, United States) and R-software version 4.0.2.^1^

## 3 Results

### 3.1 Prevalence of different bacteria causing infections in Arabian horses

As depicted in Table 1, *S. equi* was the most predominant pathogen isolated from respiratory manifestation cases (17/29; 58.6%), followed by *K. pneumoniae* and *P. aeruginosa* (9/29; 31.03%, each), *E. coli* (7/29; 24.13%), and *S. aureus* (4/29; 13.79%). The least detected pathogens were *S. pyogenes* and *C. ovis* biovar *equi* (2/29; 6.89% each), *A. baumannii*, and CoNS accounted for 1/29; 3.44%, each. A significant difference in bacterial prevalence was observed in respiratory manifestation samples (*p* < 0.0001; Table 1). Polymicrobial infections were found in 62% (18/29) of cases. *S. equi* was found in combination with *E. coli* (3/29), *K. pneumoniae* (6/29), *P. aeruginosa* (4/29), *Staphylococcus* spp. (2/29), and *C. ovis* biovar *equi* (2/29). *K. pneumoniae* was also mixed with *E. coli* and *S. pyogenes* in one case and with *S. equi* and *P. aeruginosa* in another one. Additionally, *A. baumannii* was detected in combination with *P. aeruginosa* and *S. aureus* (1/29). *S. aureus* was mixed with *P. aeruginosa* in one case and with *E. coli* in another.

**Table 1:** Number and antimicrobial resistance phenotype of different bacteria isolated from Arabian horses during the period from August 2022 to August 2023.

Clinical condition	Isolated bacteria	No. of isolated bacteria (%)^†^	*p*-value	Antimicrobial resistance phenotype			*p*- value
				MDR	XDR	PDR	
Respiratory Manifestations (*n* = 29)	*Streptococcus equi*	17 (58.6)	>0.0001^***^	–	17	–	>0.0001^***^
	*K. pneumoniae*	9 (31.03)		6	3	–	0.013^*^
	*CoNS^1^*	1 (3.44)		1	–	–	1
	*S. aureus*	4 (13.79)		4	–	–	0.006^**^
	*Streptococcus pyogenes*	2 (6.89)		–	2	–	0.2
	*P. aeruginosa*	9 (31.03)		4	5	–	0.068
	*E. coli*	7 (24.13)		4	3	–	0.115
	*A. baumannii*	1 (3.44)		–	1	–	1
	*C. ovis* biovar *equi*	2 (6.89)		–	–	–	NA
Guttural pouch empyema (*n* = 5)	*Streptococcus equi*	5	0.068	–	5	–	0.001^**^
	*K. pneumoniae*	2		2	–	–	0.2
	*S. aureus*	1		1	–	–	1
Pyogenic infections in different parts (Limbs, neck, teeth, buttocks, trunk, and hoof) (*n* = 18)	*Streptococcus equi*	1	0.005**	–	1	–	1
	*K. pneumoniae*	1		1	–	–	1
	*CoNS^1^*	3		3	–	–	0.036^*^
	*S. aureus*	9		9	–	–	>0.0001^***^
	*P. aeruginosa*	2		2	–	–	0.2
	*E. coli*	5		5	–	–	0.001^**^
	*C. ovis* biovar *equi*	6		–	–	–	NA
	*Streptococcus pyogenes*	3		–	3	–	0.036^*^
Abortion at different stages of pregnancy (3.5–7 months) (*n* = 4)	*A. baumannii*	1	0.455	–	1	–	1
	*Streptococcus equi*	1		–	1	–	1
	*K. pneumoniae*	3		3	–	–	0.036^*^
Diarrhea (*n* = 12)	*K. pneumoniae*	4	0.52	4	–	–	0.006^**^
	*E. coli*	7		7	–	–	>0.0001^***^
	*Proteus mirabilis*	4		–	4	–	0.006^**^
Ocular disorders (*n* = 3)	*Streptococcus pyogenes*	1	1	–	1	–	1
	*E. coli*	1		1	–	–	1
	*C. ovis* biovar *equi*	1		–	–	–	NA
	*K. pneumoniae*	1		1	–	–	1
	*P. aeruginosa*	1		–	1	–	1
Wound in different body parts (limbs, joints, and face) (*n* = 24)	*E. coli*	9 (37.5)	0.791	7	2	–	0.002^**^
	*Streptococcus pyogenes*	7 (29.16)		1	6	–	0.003^**^
	*Streptococcus equi*	6 (25)		–	6	–	>0.0001^***^
	*P. aeruginosa*	6 (25)		3	2	1	0.818
	*K. pneumoniae*	6 (25)		4	2	–	0.085
	*S. aureus*	10 (41.66)		10	–	–	>0.0001^***^
	*C. ovis* biovar *equi*	9 (37.5)		–	–	–	NA
Urinary tract infections (cystitis) (*n* = 1)	*E. coli*	1	NA	1	–	–	1
	*P. aeruginosa*	1		–	1	–	1
Navel ill (*n* = 2)	*S. aureus*	2	0.741	2	–	–	0.2
	*Streptococcus pyogenes*	1		–	1	–	1
	*Streptococcus equi*	1		–	1	–	1
	*E. coli*	1		1	–	–	1
	*P. aeruginosa*	1		1	–	–	1
	*C. ovis* biovar *equi*	2		–	–	–	NA
Wound between rectum and vagina (rectal tear) (*n* = 2)	*E. coli*	2	0.5	2	–	–	0.2
	*S. aureus*	1		1	–	–	1

No: number; MDR: multidrug resistant; XDR: extensively-drug resistant; PDR: pan-drug resistant; CoNS: coagulase negative staphylococci; NA: non-applicable. * *p* < 0.05; ** *p* > 0.01; *** *p* > 0.0001. †Percentage was calculated according to total number of samples and are not used where there are less than 20 observations.

*Streptococcus equi* was isolated from all foals suffering from guttural pouch empyema (GPE). *K. pneumoniae* (2/5; 40%) and *S. aureus* (1/5; 20%). Notably, both *S. equi* and *K. pneumoniae* were found in two cases and *S. equi* with *S. aureus* in other case.

*Staphylococcus aureus* was the most frequently isolated bacteria from pyogenic infections (9/18; 50%), followed by *C. ovis* biovar *equi* (6/18; 33.33%), *E. coli* (5/18; 27.78%), and CoNS and *S. pyogenes* (3/18; 16.67%, each). The least isolated bacteria were *P. aeruginosa* (2/18; 11.11%), and *S. equi* and *K. pneumoniae* (1/18; 5.56%, each). There is a highly significant difference (*p* < 0.01) in prevalence of bacteria causing pyogenic infections (Table 1).

Approximately 44.44% (8/18) of pyogenic infections were mixed infections. Notably, *E. coli* was mixed with *S. equi*, *C. ovis* biovar *equi*, *K. pneumoniae*, and CoNS in one case and found with *S. aureus* in three other cases. *C. ovis* biovar *equi* was often found in mixed infections with *S. aureus* (3 cases) and *S. pyogenes* (2 cases).

*K. pneumoniae* were isolated from cases of abortion at 6^th^ and 7^th^ months of pregnancy. Additionally, *S. equi* and *A. baumannii* were isolated from abortion at 3.5 months of pregnancy (1/4; 25%).

The most frequently isolated bacteria from diarrheic foals were *E. coli* (7/12; 58.33%), followed by *K. pneumoniae* and *P. mirabilis* (4/12; 33.33%, each). Approximately 25% (3/12) of the cases involved polymicrobial infections with *K. pneumoniae* and both *P. mirabilis* twice, and *E. coli* once.

*Streptococcus pyogenes*, *E. coli*, *C. ovis* biovar *equi*, *K. pneumoniae* and *P. aeruginosa* were isolated from the eye infection in a horse. Additionally, *S. pyogenes* and *C. ovis* biovar *equi* were identified in a case. While both *K. pneumoniae* and *P. aeruginosa* were the causative agent of ocular infection in another case. *E. coli* was the primary cause of eye infection in a horse.

*Staphylococcus aureus* exhibited the highest isolation rate in wound lesions from different body parts (10/24; 41.66%), followed by *E. coli* and *C. ovis* biovar *equi* (9/24; 37.5%, each), *S. pyogenes* (7/24; 29.16%) and *S. equi*, *P. aeruginosa*, and *K. pneumoniae* (6/4; 25%, each).

Polymicrobial wound infections were observed in 62% (15/24) of cases. A variety of bacterial combinations were identified. *E. coli*, *S. aureus*, and *S. equi* were found in two cases. *C. ovis* biovar *equi*, and *P. aeruginosa* were the causative agents in three cases. Moreover, *C. ovis* biovar *equi* and *E. coli* were found in two cases. *S. pyogenes*, *C. ovis* biovar *equi*, and *E. coli* were identified in three cases. Additionally, *E. coli* was found with *S. aureus* in three cases and with *K. pneumoniae* in one case. *S. aureus* was found with *S. equi* in two cases, and with *K. pneumoniae*, *S. equi*, *P. aeruginosa* and *C. ovis* biovar *equi* in one case. Also, *S. aureus*, *K. pneumoniae*, and *S. equi* were the primary cause of wound infections in some cases.

A single case of polymicrobial urinary tract infection (UTI) in a 21-year-old horse involved both *E. coli* and *P. aeruginosa*. Additionally, foals with navel ill (average age: 1.5 ± 0.5 months) experienced polymicrobial infections, commonly involving *S. aureus* and *C. ovis* biovar *equi*. In one case, *S. aureus* was found in mixed infections with *S. pyogenes*, *S. equi*, *E. coli*, and *P. aeruginosa*, respectively. Furthermore, swabs from rectal tear cases yielded *E. coli* and *S. aureus* isolates, with one sample exhibiting polymicrobial infections comprising both species (Table 1).

**Figure 1:**
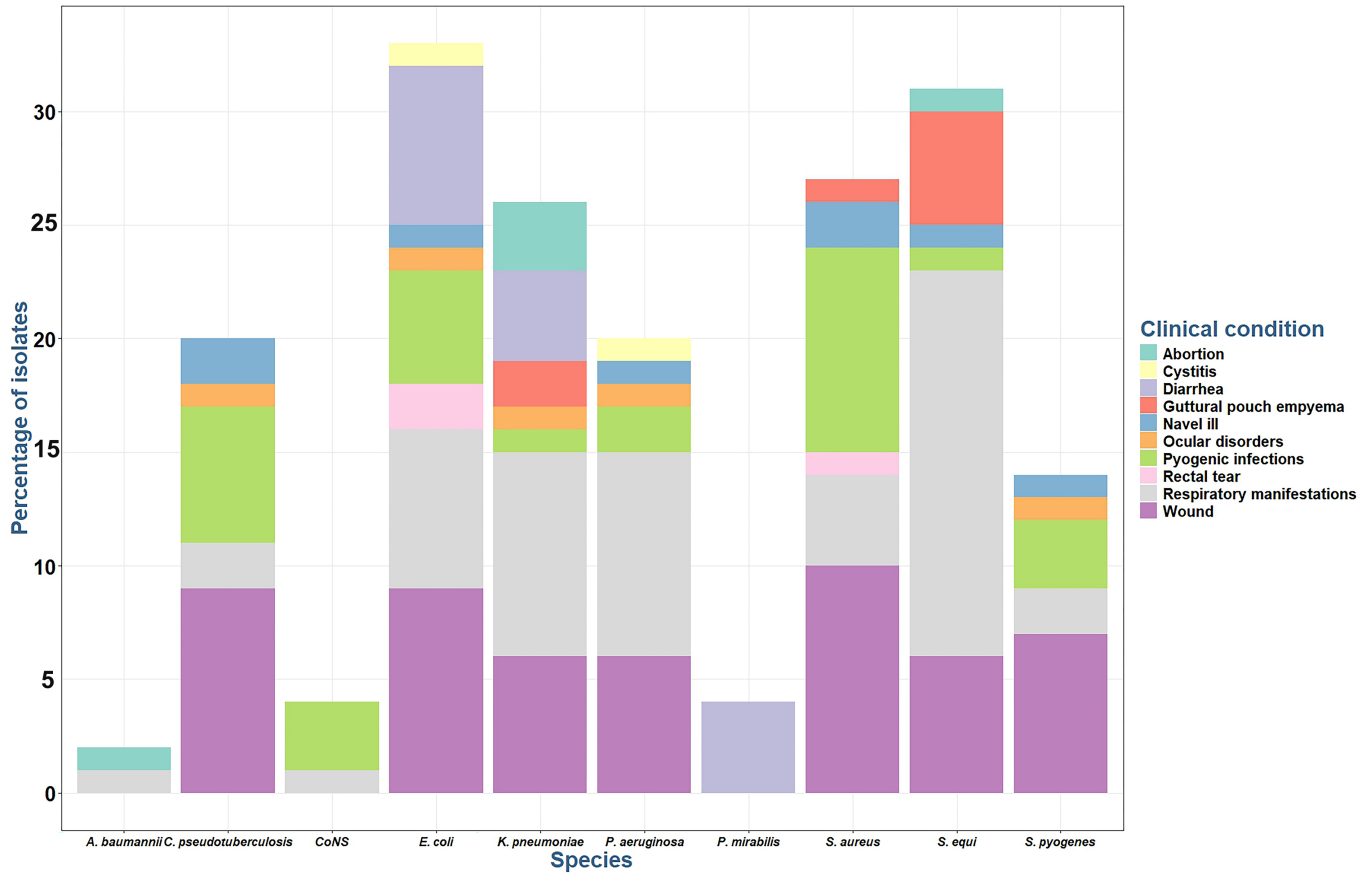
Percentage of bacterial species isolated from different infections in Arabian horses. CoNS: coagulase negative *Staphylococci*.

Among 100 cases, *Streptococcus* spp. was the most frequently isolated microorganism (45%), primarily from respiratory infections, wounds, pyogenic infections and GPE, and was less prevalent in umbilical infections, abortions, and ocular infections. *E. coli* isolates (33%) was predominantly found in wound samples, followed by respiratory, diarrheic samples, and pyogenic infections, with fewer instances in rectal tears, navel illness, UTI, and ocular infections (Figure 1).

Most *Staphylococcus* spp. isolates (31%) were recovered from wound samples, followed by pyogenic infections, and respiratory tract infections, with a minimal prevalence from foal-infected umbilicus, and GPE.

*Klebsiella pneumoniae* isolates (26%) were obtained from respiratory tract infections, followed by wound and diarrhea cases, with fewer instances in abortion and GPE cases (Figure 1). *Pseudomonas aeruginosa* isolates (20%) is primarily caused respiratory tract infections, followed by wound infections, and minimal instances of pyogenic infections, ocular, UTI, and umbilical infections. *P. mirabilis* isolates (4%) was identified exclusively in diarrheic cases. *A. baumanni* isolates (2%) was observed in abortion and respiratory tract infections.

*Corynebacterium ovis* biovar *equi* made up 20% of the cases and was most found in wound cases, followed by pyogenic infections. Its presence has been detected at a lower frequency in respiratory, navel illness, and ocular infections. All *Corynebacterium* spp. isolates were identified using direct sample PCR, and positive isolates were subjected to the amplification of narG gene for *C. ovis* biovar *equi*.

### 3.2 Emergence of extensively- and Pan-drug resistant bacteria in Arabian horses

**Figure 2:**
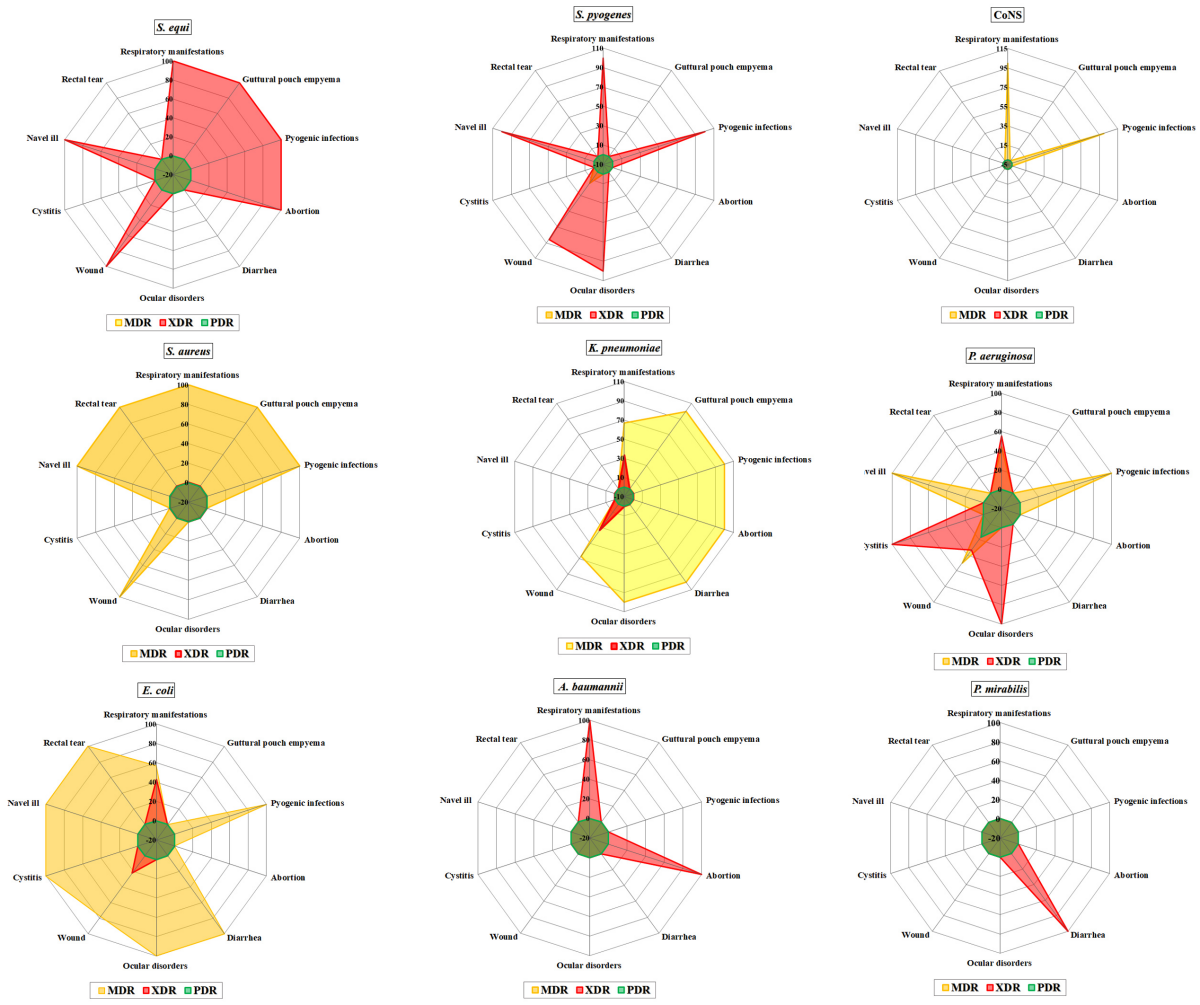
Prevalence of multi-drug resistant (MDR), extensively-drug resistant (XDR), and pan-drug resistant (PDR) bacteria from different samples.

All 161 bacterial isolates were resistant to multiple antimicrobial agents. Among the AMR isolates 91/161 (56.52%), 69/161 (42.85%) and 1/161 (0.62%) showed MDR, XDR and PDR phenotype, respectively (Figure 2).

**Figure 3:**
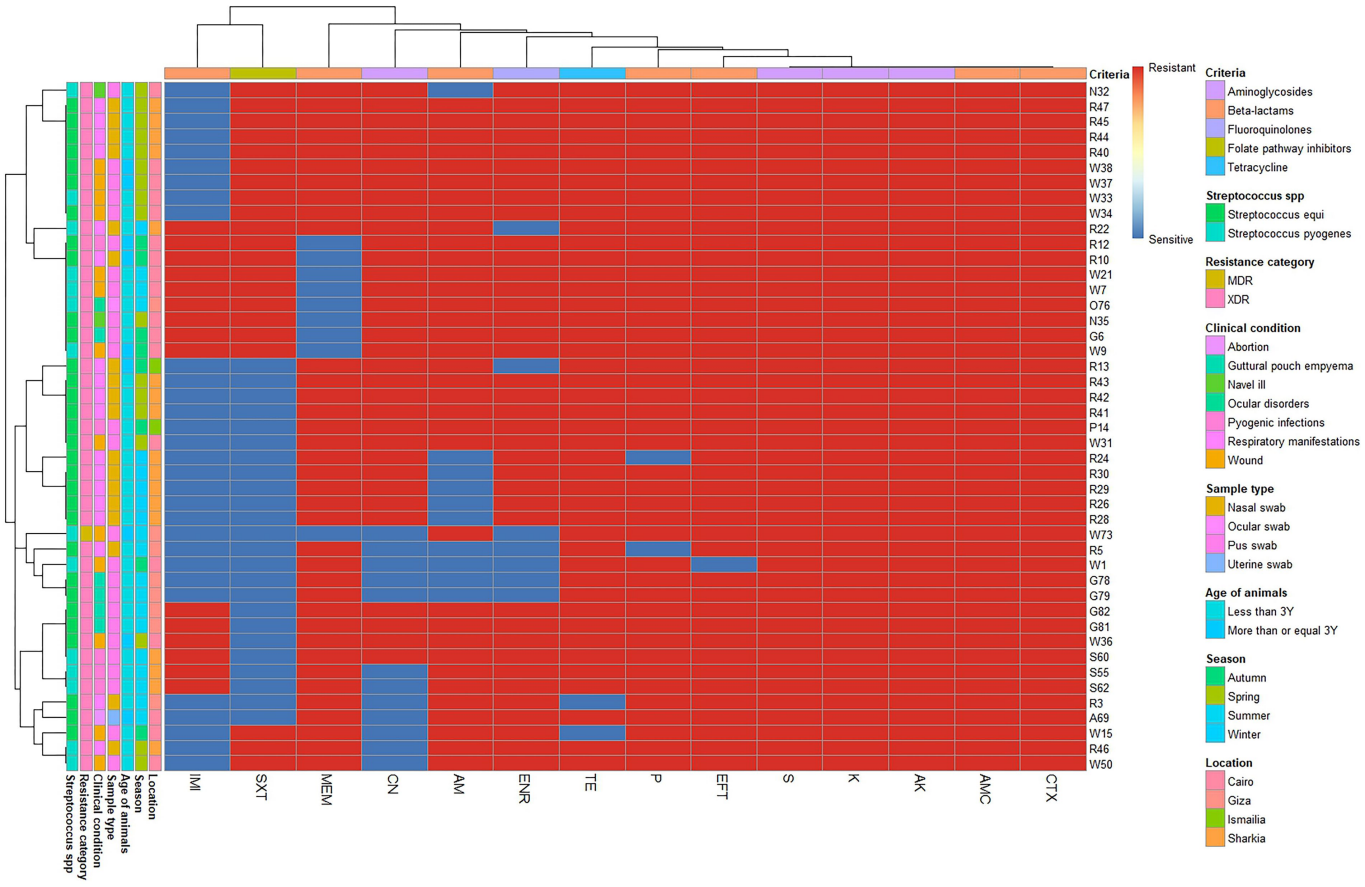
Overall distribution and clustering of *Streptococcus* species isolates under study and the patterns of their antimicrobial resistance. Different *Streptococcus* species, clinical condition, sample types, location, age of horse, season, antimicrobial classes, and resistance categories are shown for each isolate as color codes. Red and blue colors refer to the resistance/sensitivity to an antimicrobial agent. The heatmap represents the hierarchical clustering of the isolates and the antimicrobials. AM, ampicillin; CN, gentamycin; AK, amikacin; AMC, amoxicillin-clavulanic acid; IMI, imipenem; MEM, meropenem; TE, tetracycline; SXT, trimethoprim-sulfamethoxazole; P, penicillin; CTX, cefotaxime; K, kanamycin; S, streptomycin; ENR, enrofloxacin; EFT, ceftiofur.

Among *Streptococcus* spp. 44 isolates were XDR with MAR index ranging from 0.57 to 0.93. Additionally, one MDR was found in a wound case, with an MAR index of 0.64. All *Streptococcus* spp. isolates were completely resistant to amoxicillin-clavulanic acid, amikacin, kanamycin, cefotaxime, and streptomycin (Table 2; Figure 3).

**Table 2:** Resistance rates of isolated *Streptococcus* spp. to the tested antimicrobial agents and the multiple antibiotic resistance (MAR) index of the tested antimicrobials.

No. of *Streptococcus* spp. (%) (*n* = 45)					
AMA	*Streptococcus equi* (*n* = 31)	*Streptococcus pyogenes* (*n* = 14)	*p*-value	Total *Streptococcus* spp. (*n* = 45)	MAR Index^†^
AMC	31 (100)	14 (100)	NA	45 (100%)	0.07
AM	23 (74.19)	12 (85.71)	0.327	35 (77.78%)	0.06
P	29 (93.55)	14 (100)	0.47	43 (95.56%)	0.07
IMI	7 (22.58)	8 (57.14)	0.028^*^	15 (33.33%)	0.02
MEM	27 (87.09)	9 (64.29)	0.088	36 (80%)	0.06
TE	29 (93.55)	14 (100)	0.47	43 (95.56%)	0.07
ENR	27 (87.09)	11 (78.57)	0.374	38 (84.44%)	0.06
EFT	31 (100)	13 (92.86)	0.311	44 (97.78%)	0.07
SXT	12 (38.71)	9 (64.29)	0.102	21 (46.7%)	0.03
AK	31 (100)	14 (100)	NA	45 (100%)	0.07
CN	25 (80.64)	8 (57.14)	0.151	33 (73.33%)	0.05
K	31 (100)	14 (100)	NA	45 (100%)	0.07
CTX	31 (100)	14 (100)	NA	45 (100%)	0.07
S	31 (100)	14 (100)	NA	45 (100%)	0.07

AMA, antimicrobial agent; AM, ampicillin; AMC, amoxicillin-clavulanic acid; IMI, imipenem; MEM, meropenem; CN, gentamycin; AK, amikacin; TE, Tetracycline; SXT, trimethoprim-sulphamethoxazole; ENR, enrofloxacin; EFT, ceftiofur; P, penicillin; K, kanamycin; CTX, cefotaxime; S, streptomycin. ^†^MAR index for each antimicrobial agent = total number of resistance isolate to antimicrobial agent /total number of antimicrobials tested × total number of isolates (27). NA: non-applicable; * *p* < 0.05; ** *p* < 0.01; *** *p* < 0.0001.

Furthermore, *S. pyogenes* showed complete resistance to penicillin and tetracycline, followed by ceftiofur (92.86%), ampicillin (85.71%), enrofloxacin (78.57%), meropenem, and trimethoprim-sulfamethoxazole (64.29% each), as well as imipenem and gentamycin (75.14% each). In contrast, *S. equi* showed complete resistance to Ceftiofur, followed by penicillin and tetracycline (93.55%), enrofloxacin and meropenem (78.10%), gentamycin (80.65%), and ampicillin (74.19%). However, the highest sensitivity was observed for imipenem (77.42%), followed by trimethoprim-sulfamethoxazole (61.29%). Additionally, there was a significant difference in the resistance of both *Streptococcus* spp. to imipenem (*p =* 0.028; Table 2).

All *Staphylococcus* spp. displayed MDR pattern with MAR index range (0.3–0.8). CoNS exhibited complete resistance to fosfomycin, followed by cefoxitin, fusidic acid, clindamycin, erythromycin, and tetracycline (75% each), and Quinupristin-dalfopristin and doxycycline (50%, each). However, high sensitivity was observed for tigecycline, ceftaroline, teicoplanin, vancomycin, ciprofloxacin, and linezolid, followed by gentamycin, rifampin, trimethoprim-sulfamethoxazole, and chloramphenicol (75% each) (Table 3; Supplementary Figure S2).

**Table 3:** Resistance rates of isolated *Staphylococcus* spp. to the tested antimicrobial agents and the multiple antibiotic resistance (MAR) index of the tested antimicrobials.

AMA	No. of *Staphylococcus* spp. (%) (*n* = 31)				
	*S. aureus* (*n* = 27)	CoNS (*n* = 4)	*p*-value	Total *Staphylococcus* spp. (*n* = 31)	MAR Index^†^
CN	8 (29.62)	1 (25)	0.673	9 (29.03%)	0.02
RA	21 (77.77)	1 (25)	0.063	22 (70.97%)	0.04
CPT	3 (11.11)	0	0.651	3 (9.68%)	0.005
FOX	26 (96.29)	3 (75)	0.245	29 (93.55%)	0.05
CIP	11 (40.74)	0	0.154	11 (35.48%)	0.02
SXT	16 (59.25)	1 (25)	0.228	17 (54.84%)	0.03
FA	22 (81.48)	3 (75)	0.598	25 (80.65%)	0.04
TEC	1 (3.7)	0	0.871	1 (3.23%)	0.002
VA	1 (3.7)	0	0.871	1 (3.23%)	0.002
TGC	0	0	NA	0 (0%)	0.00
DA	26 (96.29)	3 (75)	0.245	29 (93.55%)	0.05
E	22 (81.48)	3 (75)	0.598	25 (80.65%)	0.04
LNZ	1 (3.7)	0	0.871	1 (3.23%)	0.002
SYN	25 (92.59)	2 (50)	0.07	27 (87.10%)	0.05
FF	27 (100)	4 (100)	NA	31 (100%)	0.06
TE	22 (81.48)	3 (75)	0.598	25 (80.65%)	0.04
DO	9 (33.33)	2 (50)	0.447	11 (35.48%)	0.02
C	18 (66.67)	1 (25)	0.149	19 (61.29%)	0.03

AMA, antimicrobial agent; CN, gentamycin; RA, rifampin; CPT, ceftaroline; FOX, cefoxitin; CIP, ciprofloxacin; SXT, trimethoprim-sulphamethoxazole; FA, fusidic acid; TEC, teicoplanin; VA, vancomycin; TGC, tigecycline; DA, clindamycin; E, erythromycin; LNZ, linezolid; SYN, quinupristindalfopristin; FF, Fosfomycin; TE, tetracycline; DO, doxycycline; C, chloramphenicol. ^†^MAR index for each antimicrobial agent = total number of resistance isolate to antimicrobial agent /total number of antimicrobials tested × total number of isolates (27). NA: non-applicable; * *p* < 0.05; ** *p* < 0.01; *** *p* < 0.0001.

On the counterpart, *S. aureus* exhibited complete resistance to fosfomycin followed by clindamycin and cefoxitin (96.3% each), Quinupristin-dalfopristin (92.59%), tetracycline, fusidic acid, and erythromycin (81.48% each), rifampin (77.78%), chloramphenicol (66.67%), and trimethoprim-sulfamethoxazole (59.26%). In contrast, the isolates showed complete susceptibility to tigecycline, followed by teicoplanin, vancomycin, linezolid (96.3%), ceftaroline (88.89%), gntamycin (70.37%), doxycycline (66.67%), and ciprofloxacin (59.2%). There were no statistically significant differences (*p* > 0.05) in the resistance profiles among all *Staphylococcus* spp. isolates for all tested antimicrobials (Table 3).

Methicillin resistance was determined for *Staphylococcus* spp. based on their phenotypic resistance to cefoxitin. Of the 27 *S. aureus* isolates, 26 (96.3%) were methicillin-resistant *S. aureus* (MRSA). All MRSA isolates were MDR and susceptible to tigecycline. Additionally, 75% of CoNS were considered methicillin-resistant CoNS (MRCoNS). MIC_50_ and MIC_90_ of vancomycin for *Staphylococci* spp. were 0.5 and 2 μg/mL, respectively. Only one isolate was found to be VRSA (MIC = 16 μg/mL). VRSA also showed resistance to ceftaroline and cefoxitin. All *Staphylococcus* spp. isolates were susceptible to tigecycline, with MIC_50_ and MIC_90_ values of 0.25 μg/mL and 0.5 μg/mL, respectively.

**Figure 4:**
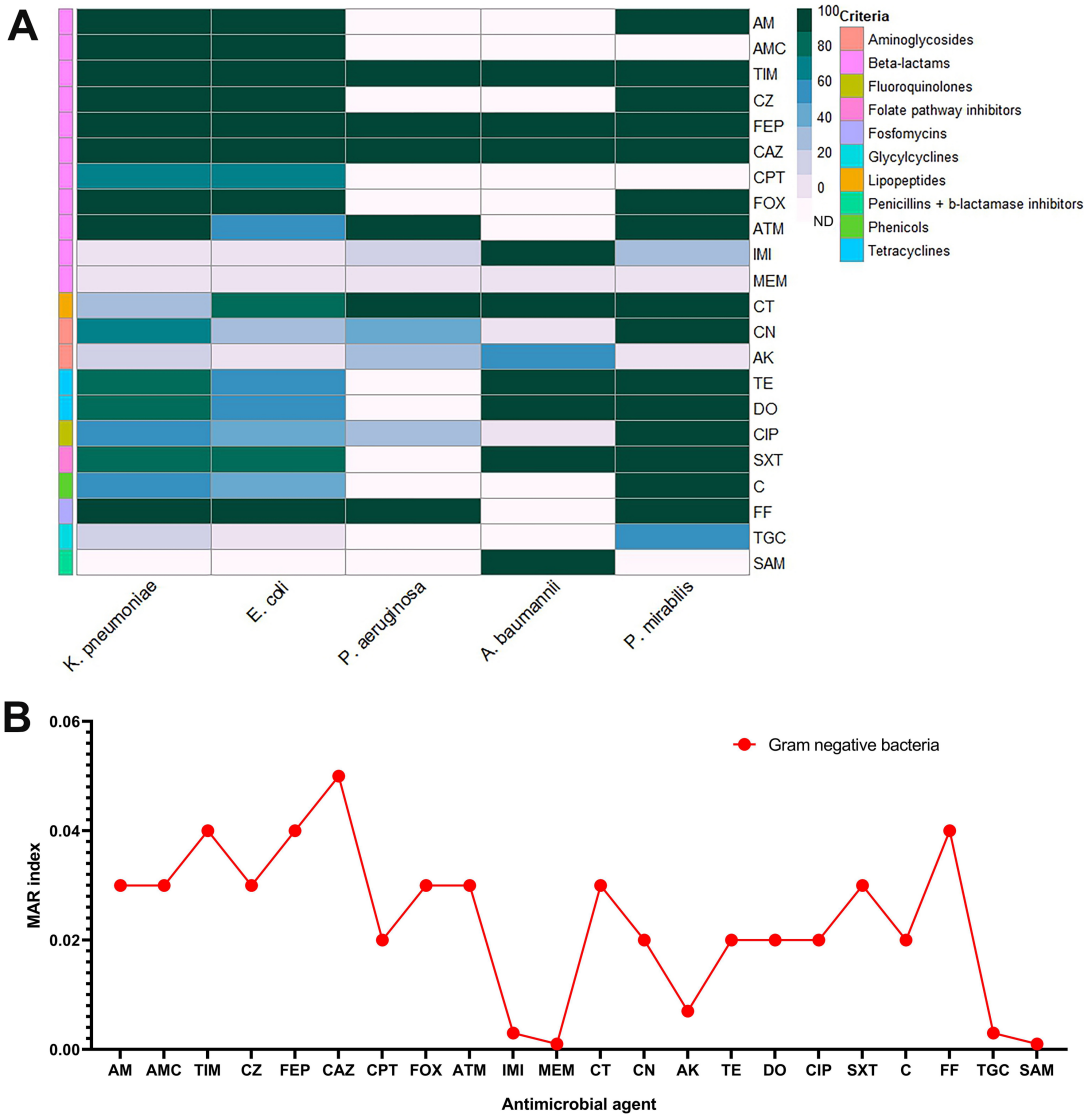
Resistance rates of the recovered Gram-negative bacteria against the tested antimicrobial agents **(A)** and multiple antibiotic resistance (MAR) index of the tested antimicrobials. **(B)**. AM, ampicillin; CN, gentamycin; AK, amikacin; TIM, ticarcillin-clavulanic acid; AMC, amoxicillin-clavulanic acid; CZ, cefazolin; FEP, cefepime; CAZ, ceftazidime; FOX, cefoxitin; ATM, aztreonam; IMI, imipenem; MEM, meropenem; CT, colistin; TE, tetracycline; DO, doxycycline; SXT, trimethoprim-sulfamethoxazole; C, chloramphenicol; FF, Fosfomycin; TGC, tigecycline; CIP, ciprofloxacin; SAM, ampicillin-sulbactam; CPT, ceftaroline.

Out of 63 *Enterobacteriaceae* isolates 49 isolates showed MDR pattern with MAR index range (0.4–0.8) and 14/63 isolates showed XDR pattern with MAR index range (0.8–0.9). They exhibited total resistance amoxicillin-clavulanic acid AMC, cefazolin, cefepime, ceftazidime, and fosfomycin. Statistically significant differences were observed in the prevalence of antimicrobial resistance among Gram-negative isolates (Supplementary Table S3). The resistance rates of all isolated Gram-negative bacteria to the tested antimicrobial agents are shown in Figure 4.

Among *E. coli* isolates 84.85% (28/33) were MDR and 15.15% (5/33) were XDR. *E. coli* isolates showed complete resistance to amoxicillin-clavulanic acid, cefazolin, cefepime, ceftazidime and fosfomycin, high resistance to ampicillin (96.9%), followed by ticarcillin-clavulanic acid (93.9%), cefoxitin (90.9%), colistin (84.8%), trimethoprim-sulfamethoxazole (75.7%), ceftaroline (66.6%), tetracycline and doxycycline (51.5%, each), aztreonam (48.4%), and chloramphenicol (42.4%). The total sensitivity was observed for tigecycline, imipenem and meropenem, followed by amikacin (93.9%), gentamycin (78.8%) and ciprofloxacin (60.7%).

A high proportion of *K. pneumoniae* isolates were found to be resistant to multiple drugs, with 80.77% being MDR and 19.23% (5/26) being XDR. The isolates exhibited high levels of resistance to amoxicillin-clavulanic acid, cefazolin, cefepime, ceftazidime and fosfomycin, ampicillin, cefoxitin and ticarcillin-clavulanic acid followed by aztreonam (96.2%), doxycycline, tetracycline, and trimethoprim-sulfamethoxazole (76.9%, each), ceftaroline (73.1%), gentamycin (61.5%), chloramphenicol (57.7%), ciprofloxacin (53.8%), and colistin (30.8%). Conversely, the isolates demonstrated susceptibility to imipenem and meropenem, with moderate susceptibility observed for tigecycline (84.6%) and amikacin (80.8%).

All *P. mirabilis* isolates showed XDR pattern. They demonstrated complete resistanceto amoxicillin-clavulanic acid, cefazolin, cefepime, ceftazidime and fosfomycin, ticarcillin-clavulanic acid, ampicillin, tetracycline, doxycycline, ciprofloxacin, trimethoprim-sulfamethoxazole, colistin, gentamycin, aztreonam, cefoxitin and ceftaroline and moderate resistance rate for tigecycline (50%). Amikacin and meropenem demonstrated complete sensitivity, followed by imipenem with 75% sensitivity.

Among *P. aeruginosa* isolates 50% were MDR with MAR index ranging from 0.4–0.6. Nine isolates (45%) were XDR with MAR index of 0.6–0.8 and one isolate was PDR with MAR index of 1.0. All isolates exhibited resistance to ticarcillin-clavulanic acid, ceftazidime, colistin, and Fosfomycin (100%), followed by aztreonam and cefepime (90%, each), gentamycin (40%), and ciprofloxacin (30%). The highest sensitivity (95%) was found for meropenem, followed by imipenem (85%) and amikacin (75%).

Both *A. baumannii* isolates showed XDR pattern with MAR index ranging from 0.7–0.8. They were completely resistant to ticarcillin-clavulanic acid, cefepime, ceftazidime, imipenem, colistin, tetracycine, doxycycline, trimethoprim-sulfamethoxazole, ampicillin-sulbactam. They demonstrated complete susceptibility for gentamicin, ciprofloxacin, and meropenem. Followed by AK (50%).

Testing MIC values of tigecycline for 63 *Enterobacteriaceae* isolates, revealed that 33 *E. coli*, 22 *K. pneumoniae*, and 2 *P. mirabilis* were susceptible to tigecycline. The remaining six isolates, including four *K. pneumoniae* and two *P. mirabilis*, were resistant to tigecycline. The tigecycline MIC_50_ and MIC_90_ values for *E. coli* isolates were 0.25 μg/mL and 1 μg/mL, respectively. For *K. pneumoniae* isolates, these values were 0.5 μg/mL and 16 μg/mL, respectively. Additionally, tigecycline MIC range for *P. mirabilis* was 0.0625–8 μg/mL.

Among 85 tested Gram-negative bacteria, there was only 28 *E. coli*, 8 *K. pneumoniae,* 20 *P. aeruginosa,* 4 *P. mirabilis*, and 2 *A. baumannii* (72.94%) exhibited colistin resistance. The remaining 23 isolates (27.06%), including 18 *K. pneumoniae* and 5 *E. coli* isolates were considered colistin-susceptible.

The colistin MIC_50_ and MIC_90_ for *E. coli* isolates, were 4 μg/mL and 64 μg/mL, respectively. For *K. pneumoniae* isolates, were 0.5 μg/mL and 16 μg/mL, respectively. In *P. aeruginosa* isolates the MIC_50_ was 32 μg/mL and the MIC_90_ was 64 μg/mL. The MIC range for *P. mirabilis* isolates was 16–64 μg/mL, whereas that for *A. baumannii* was 64 μg/mL.

### 3.3 Isolation and characterization of lytic phages

**Figure 5:**
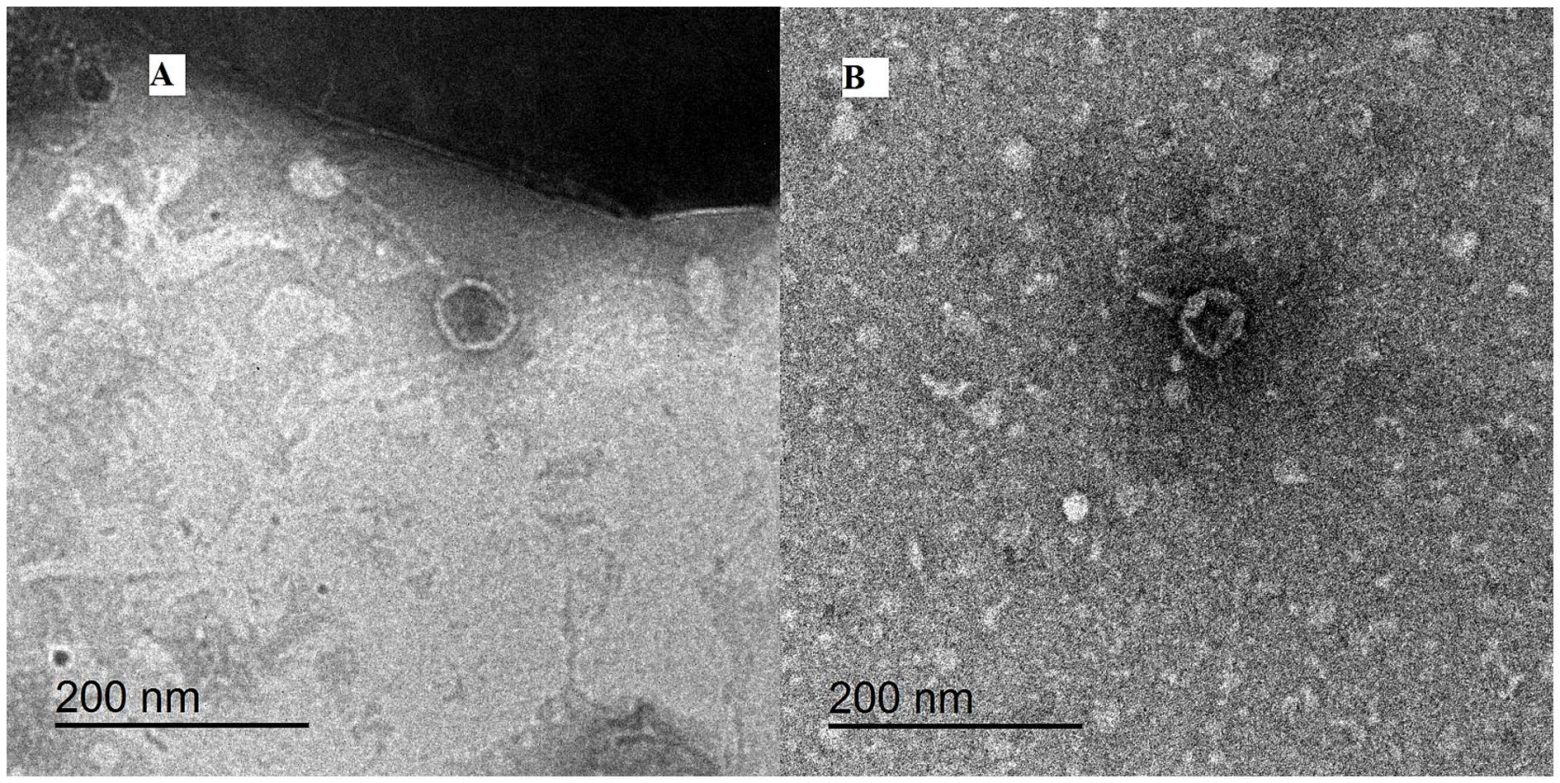
The morphology of phages under transmission electron microscope negatively stained with uranyl acetate. **(A)** vB_Pae_LS225; **(B)** vB_Pae_LP125. The scale bar = 200 nm.

Two lytic phages (vB_Pae_LP125 and vB_Pae_LS225) were isolated based on the spot and plaque assays targeting host bacteria (*P. aeruginosa*). The plaque morphology of the two isolated phages showed various sizes, with a clear plaque center and translucent surrounding area. vB_Pae_LP125 produced small single clear plaques (1 mm), while vB_Pae_LS225 produced medium size single clear plaques (2.5 mm). Based on TEM micrographs obtained from high-titre lysates, phage vBPaeLP125 exhibited a hexagonal head approximately 50 nm in diameter and a short tail measuring approximately 34 nm, consistent with podovirus-like morphology. Phage vBPaeLS225 displayed an isometric head of approximately 66 nm and a long (103 nm), flexible, non-contractile tail, resembling siphovirus-like morphology (Figures 5A,B).

### 3.4 Phage DNA extraction and restriction enzyme analysis

**Figure 6:**
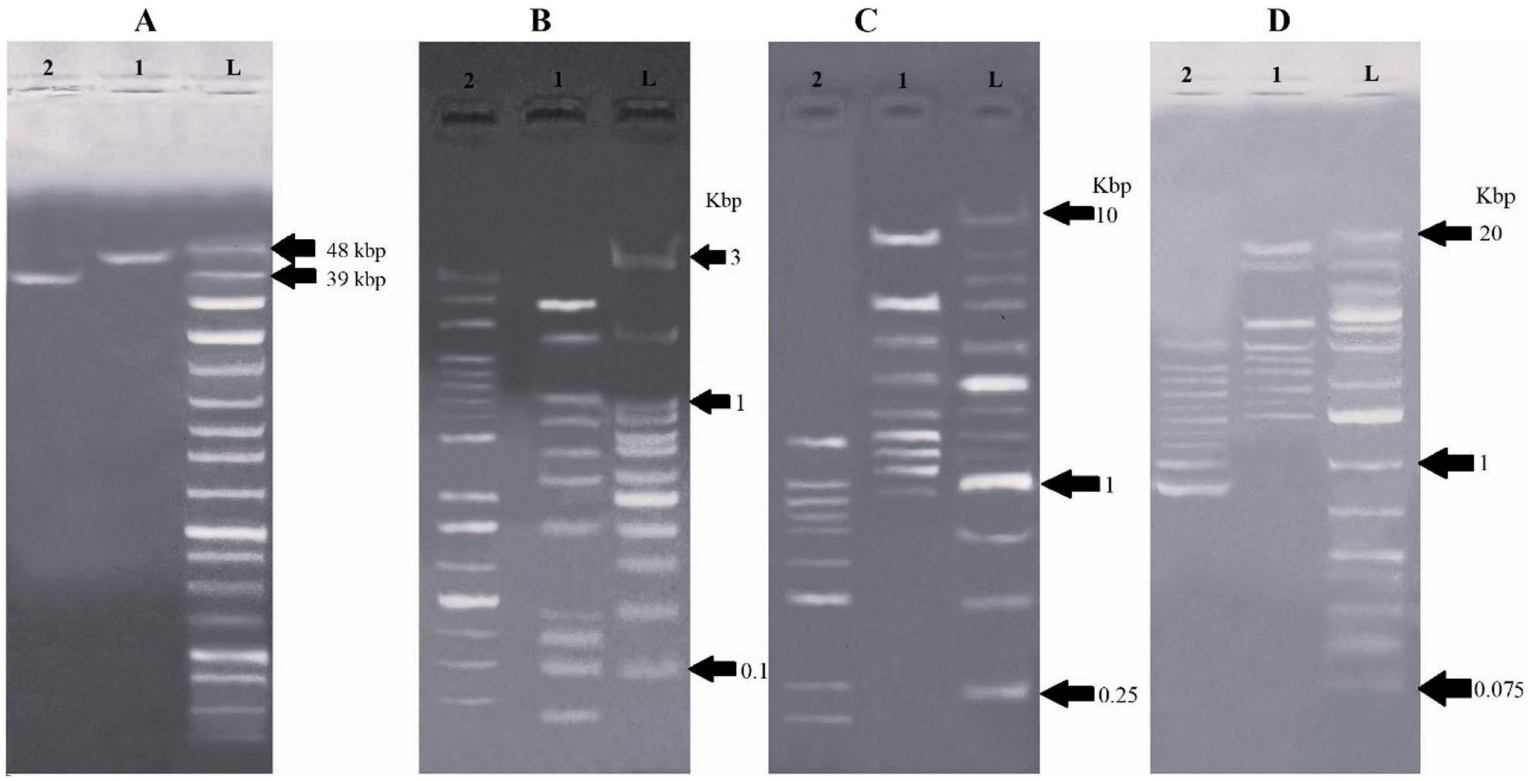
The restriction endonuclease analysis of genomic DNA of phages on agarose gel electrophoresis. **(A)** Undigested genomic DNA. Lane L: ladder (250 bp-48502 bp), lane 1: DNA of vB_Pae_LP125, lane 2: DNA of vB_Pae_LS225; **(B)** The LP125 and LS225 DNA digested with *hinfI*. Lane L: ladder (0.1–3kbp), 1: LP125 *hinfI* digestion, 2: and LS225 *hinfI* digestion; **(C)** The LP125 and LS225 DNA digested with *haeIII*. Lane L: ladder (0.25–10kbp), 1: LP125 *haeIII* digestion, 2: and LS225 *haeIII* digestion; **(D)** The LP125 and LS225 DNA digested with *hindIII*. Lane L: ladder (0.075–20kbp), 1: LP125 *hindIII* digestion, 2: and LS225 *hindIII* digestion.

Both phages had double-stranded linear DNA (dsDNA) and the genomic size for vB_Pae_LP125 is ~48 kbp and vB_Pae_LS225 is ~39 kbp (Figure 6A). Both phages DNA produced restriction banding patterns after *hinfI*, *haeIII* and *hind III* digestion (Figures 6B–D).

### 3.5 Host range of isolated phages

Table 4 showed the host range and the titer for each phage exhibited as PFU/ml. The host range for each phage was determined against *P. aeruginosa*, *E. coli*, *K. pneumoniae*, *S. aureus*, *P. mirablis*, *A. baumannii*, *S. equi*, and *S. pyogenes* strains by spot assay, then confirmed by plaque assays. Plaque morphologies of phage infections on semi-solid agar plates revealed host-specific plaque formation. There were distinct differences in plaque characteristics, including variations in size (small or large plaques) and morphology (clear, circular plaques or turbid, irregular plaques) (Supplementary Figures S3, S4).

**Table 4:** Titers and lytic spectra of bacteriophages vB_Pae_LP125 and vB_Pae_LS225 obtained on different bacterial strains.

Tested species	Isolate code no.	Lytic area			
		vB_Pae_LS225		vB_Pae_LP125	
		Spot test	Titer (PFU/ml)	Spot test	Titer (PFU/ml)
*Pseudomonas aeruginosa*	E77	+++	1.5 ×10^8^	+++	2.2 ×10^9^
	E8	+++	2.0 ×10^7^	−	−
	E10	+++	1.1 × 10^7^	+++	6.1 ×10^8^
*Klebsiella pneumoniae*	E11	+	2.9×10^7^	−	−
	E46	−	−	+++	7.3×10^6^
	E73	−	−	+	2.3×10^5^
*Staphylococcus aureus*	E18	+	8.1×10^7^	+++	1.3×10^7^
	E38	+	1.1 ×10^5^	+++	6.2×10^6^
	E98	−	−	+	3.3×10^6^
*Escherichia coli*	E48	+	6.0×10^6^	−	−
	E70	+++	3.1 ×10^5^	+	5.2×10^6^
	E42	+++	1.7×10^4^	+	1.2×10^5^
*Streptococcus equi*	E38	+	3.0×10^6^	+++	6.7×10^7^
	E5	−	−	−	−
*Streptococcus pyogenes*	E22	+	5.2×10^5^	+++	1.1×10^6^
*Proteus mirabilis*	E83	+++	2.8×10^5^	+	3.3×10^6^
	E16	+	4.0×10^4^	+	1.8×10^5^
	E85	+	1.0×10^3^	−	−
*Acinetobacter baumannii*	E69	−	−		−
	E53	+	8.0×10^5^		−

+++: The isolates were susceptible to phage and produce clear plaques with titers (PFU/ml), +: The isolates were susceptible to phage and produce turbid plaques with titers, and −: No plaques were produced.

### 3.6 Heat, ultraviolet and pH stability of phages

**Figure 7:**
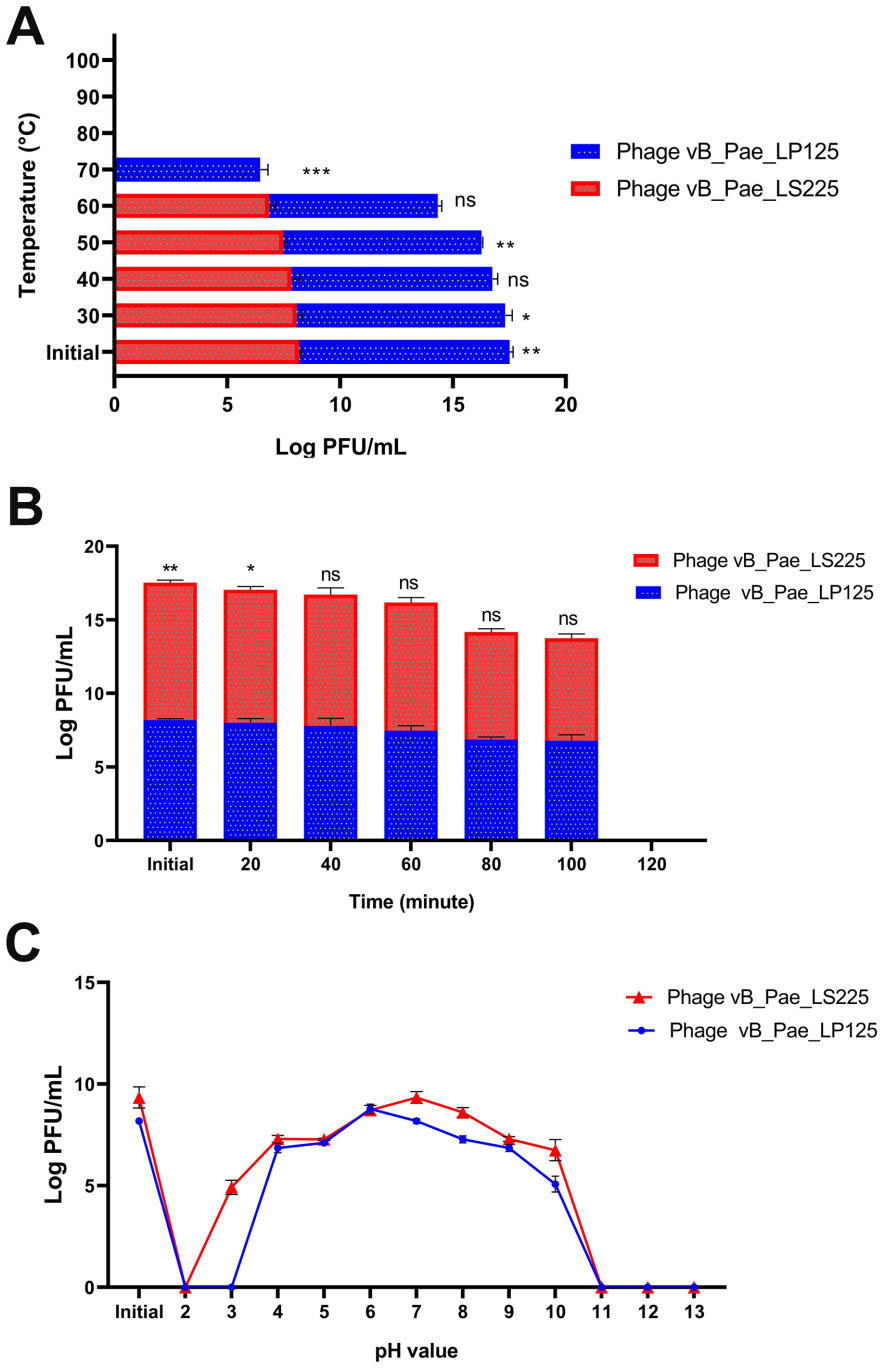
Phage stability studies; **(A)** Sensitivity of the isolated phages (vB_Pae_LP125 and vB_Pae_LS225) to various temperatures; **(B)** Effect of time exposure to U. V. irradiation on survival of phages isolates; **(C)** Effect of pH values on different phages isolates. The results were expressed as mean ± standard error. PFU: plague forming unit. ns: non-significant variations between the two phages at each point. *, **, *** indicates significant differences between the effect of two phages at each point using independent sample T-test; * *p* < 0.05, * *p* < 0.01, ** *p* < 0.001.

Both phages were considered thermostable at temperatures ranging from 30 to 60 °C. However, phage inactivation was detected after exposure to 70 °C for 10 min for phage vB_Pae_LP125 and 80 °C for phage vB_Pae_LS225, rendering them ineffective (Figure 7A). There were significant differences (*p* < 0.0001) in the effect of different temperatures on the survival of vB_Pae_LP125, and vB_Pae_LS225 with the highest effect at the initial temperature, 30 °C, and 40 °C for both phages (Supplementary Table S7).

Similarly, the exposure of both phages to UV irradiation for more than 120 min rendered them inactive and lost their infectivity (Figure 7B). There were significant differences (*p* < 0.0001) in the effect of different time exposure to U. V. irradiation on survival of vB_Pae_LP125, and vB_Pae_LS225 phages isolates with the highest effect at the initial time and after 20, 40, and 60 min for both phages (Supplementary Table S8).

The pH stability of the phage was determined at various pH values for 24 h. Phages were highly stable at pH 7; therefore, the phages were considered neutral. Increasing the pH above 10 and decreasing it to below 4 revealed no plaque formation activity. The highest number of plaques were observed at pH 7, which were sustained up to pH 10 (Figure 7C). There were significant differences (*p* < 0.0001) in the effect of different pH on the survival of vB_Pae_LP125, and vB_Pae_LS225 phages isolates with the highest effect at the initial pH and pH 6, and 7 for both phages (Supplementary Table S9).

### 3.7 Adsorption of the phage to the host cell and one-step growth curve

**Figure 8:**
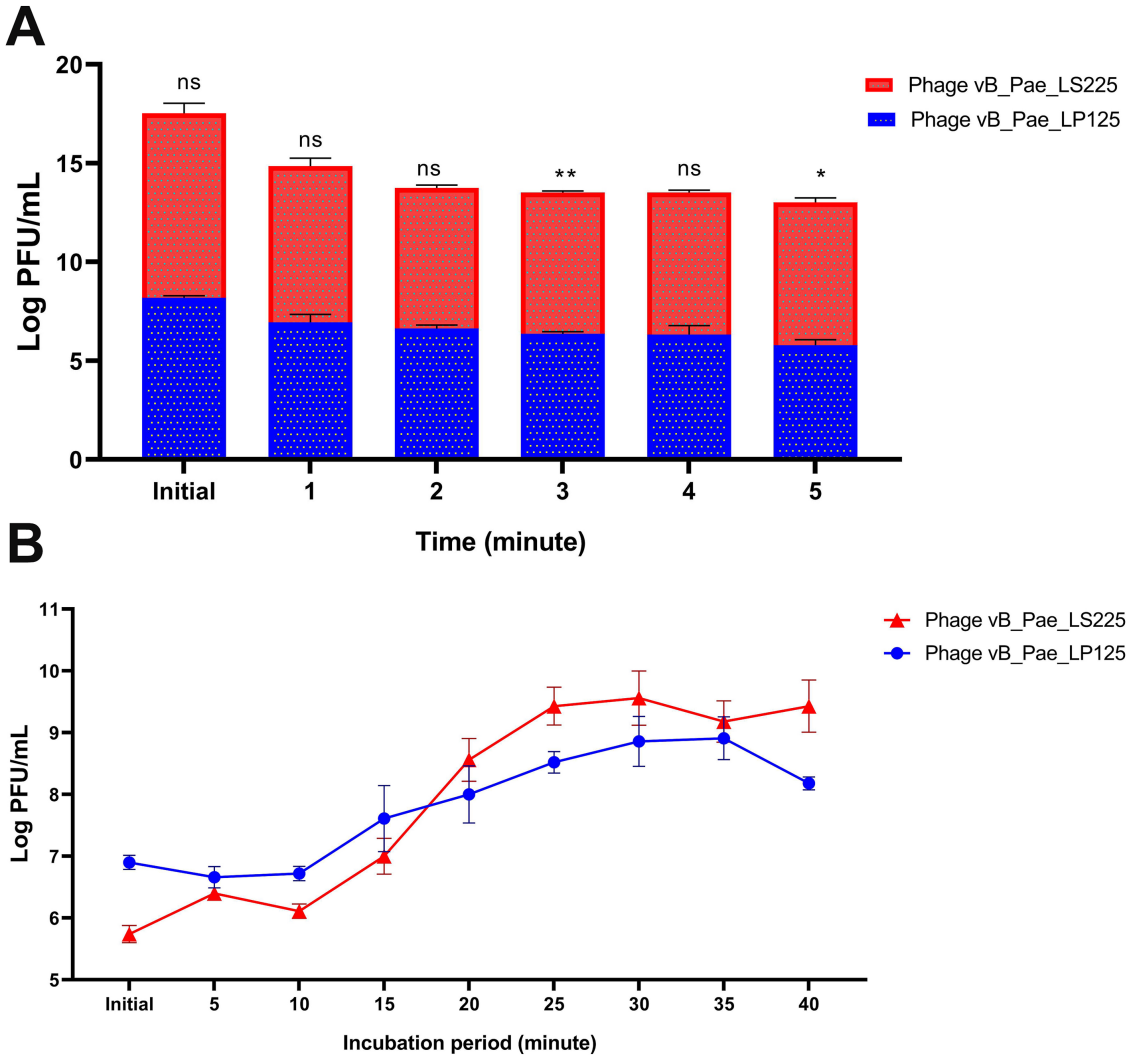
Adsorption rate **(A)**, and one-step growth **(B)** of the isolated phages (vB_Pae_LP125 and vB_Pae_LS225). The results were expressed as mean ± standard error. PFU: plague forming unit. ns: non-significant variations between the two phages at each point. *, ** indicates significant differences between the effect of two phages at each point using independent sample T-test; * *p* < 0.05, * *p* < 0.01.

Both phages demonstrated effective adsorption durations when tested against *P. aeuriginosa* strain (host strain), as shown in Figure 8A. The adsorption rate constants were 99.3% for vB_Pae_LP125 after 4 min, and 99.9% for vB_Pae_LS225 after 3 min. There were significant differences (*p* < 0.05) in the effect of different adsorption durations of vB_Pae_LP125, and vB_Pae_LS225 phages isolates with the highest effect at the initial time for both phages (Supplementary Table S10).

In the one-step growth assay using the host strain, the latent period for vB_Pae_LP125 and vB_Pae_LS225 were approximately 10 min, with corresponding burst sizes of 120 PFU/cell and140 PFU/cell, respectively. Conversely, the rise periods for vB_Pae_LP125 and vB_Pae_LS225 were 15 min, as illustrated in Figure 8B. There were significant differences (*p* < 0.0001) in the effect of different incubation periods of vB_Pae_LP125, and vB_Pae_LS225 with the highest effect after 25, 30, and 35 min for both phages (Supplementary Table S11).

### 3.8 Bacteriophage cocktails to treat infected wounds in Arabian horses

**Figure 9:**
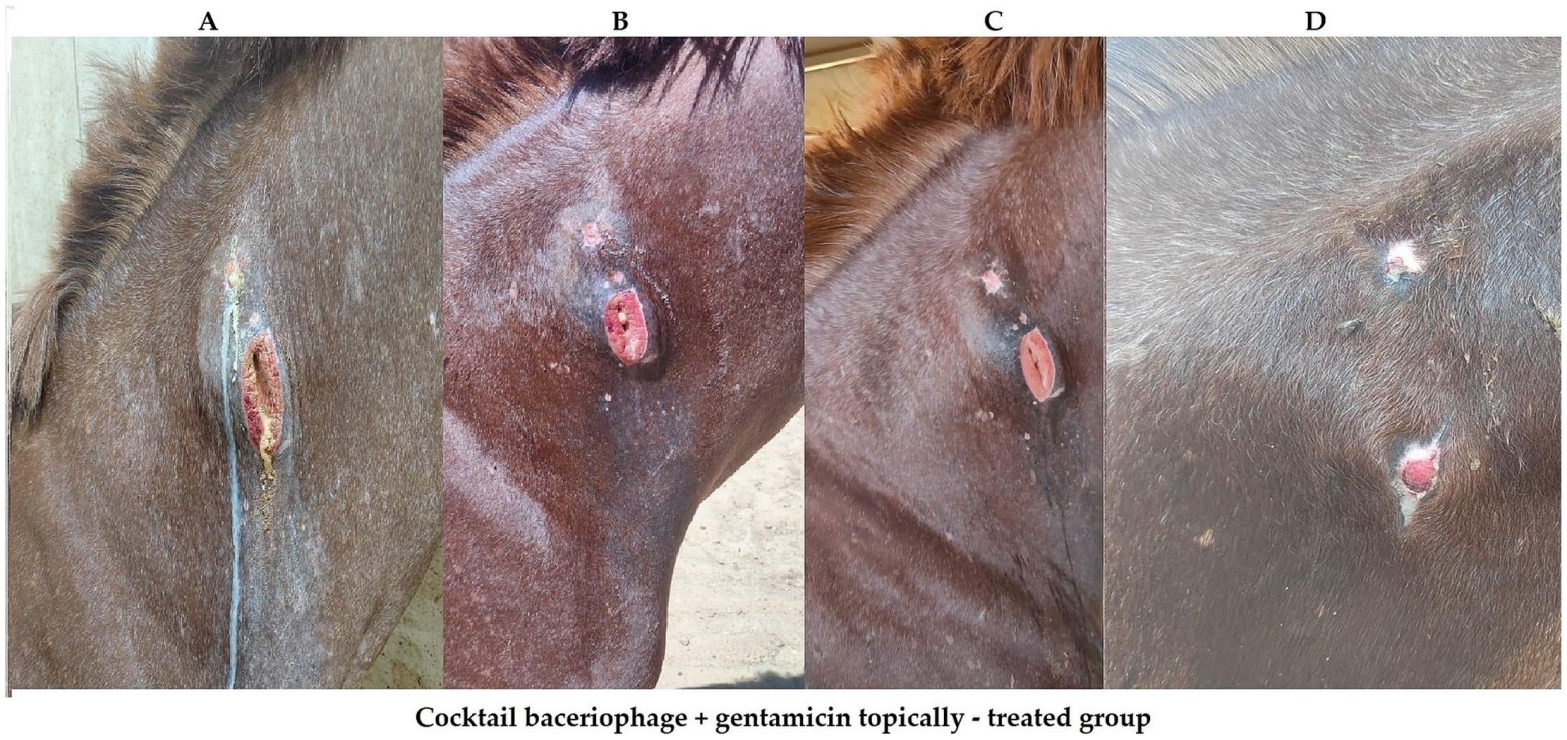
Panels **(A-D)** show the progression of wound healing on Arabian horse’s skin treated with a cocktail of bacteriophage and gentamicin. Panel **(A)** displays the initial wound with discharge. Panel **(B)** shows partial healing with reduced discharge at third day of treatment. Panel **(C)** reveals further improvement with the wound partially closed at seventh day of treatment. Panel **(D)** depicts significant healing with almost closed wound at day 14 of treatment.

**Figure 10:**
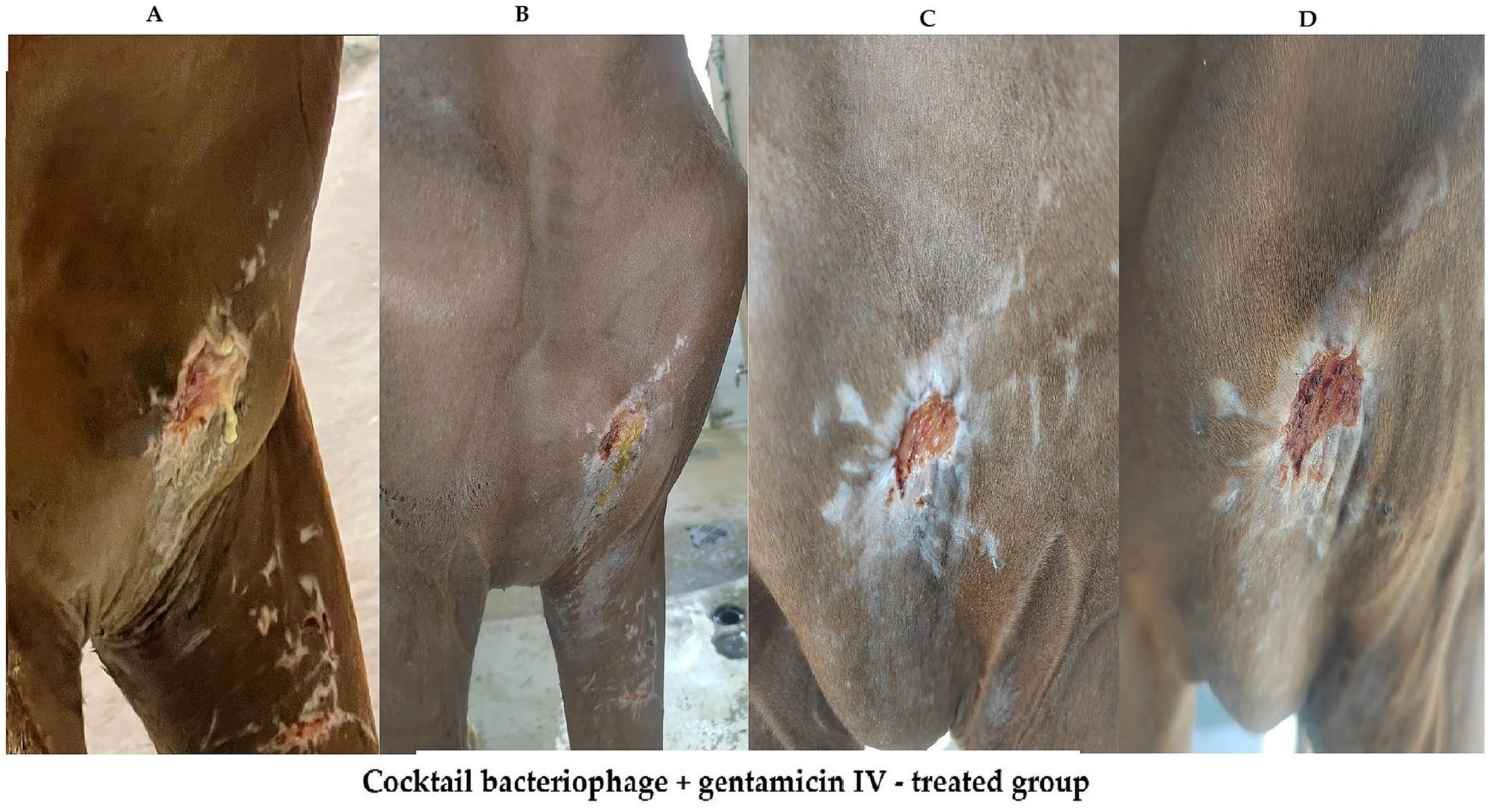
Series of four images labeled **(A-D)**, showing the healing progression of an infected wound on the skin of Arabian horse treated with bacteriophage cocktail and gentamicin intravenous (IV). Image **(A)** shows the initial large, inflamed wound. Images **(B-D)** display gradual healing, with reduced inflammation and wound size at days 3, 7, and 14 post treatment.

**Figure 11:**
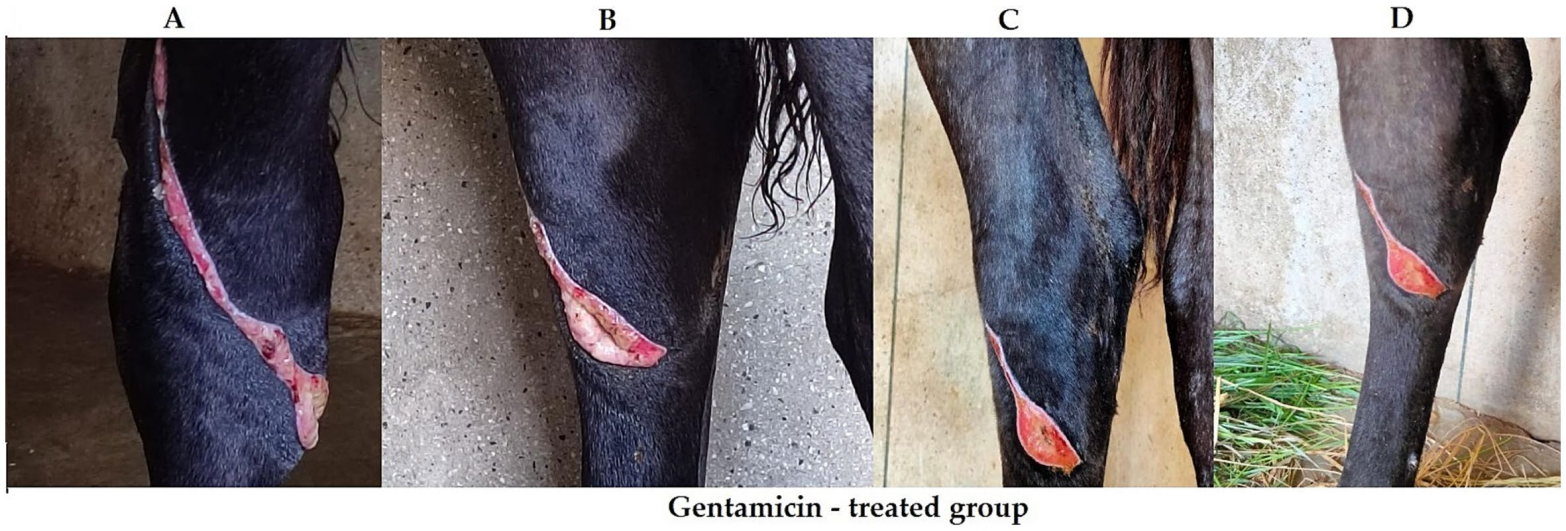
Progression of wound healing in a gentamicin-treated group. Panel **(A)** shows the initial wound. Panel **(B)** has slight improvement at day 3 post treatment. Panel **(C)** shows further healing progress at day 7 post treatment, and Panel **(D)** displays healing at day 14 post treatment.

Upon admittance for therapy, four horses received antibiotic treatment (gentamicin), while the other eight horses received a combination of a bacteriophage cocktail (vB_Pae_LP125 and vB_Pae_LS225) and gentamicin for treatment wound infections (Figures 9–11). Bacteriological examination of wound lesions in the enrolled horses revealed the presence of *S. aureus* or *P. aeruginosa*. Complete clearance of these pathogens was observed after the treatment course. Wound evaluations were conducted after 3–5 days to assess the treatment’s effectiveness, revealing a reduction in exudate and signs of inflammation in all horses. By day 7–10 of treatment, the formation of a granulation tissue was noted, and by 12–14 days of treatment, the formation of scars was observed (Figures 9–11). Further treatment involved the use of steroid anti-inflammatory medications to decrease the size of the scar tissue. There were significant differences (*p* < 0.05) in the wound closure % among the gentamicin group and phage cocktail+gentamicin groups on days 3, 5, 7, 10, and 14. The highest significant wound closure % was detected among the phage cocktail+gentamicin injection group on days 3, 5, 7, and 10. On days 12 and 14, the highest wound closure% were detected among phage cocktail+gentamicin groups, with no significant difference between the two groups (Supplementary Figure S5).

## 4 Discussion

Antimicrobial resistance is a growing global health crisis. As existing treatments become less effective, new approaches are urgently needed. This study investigated the frequency of equine infections caused by antibiotic resistant bacteria and explored the potential of phage therapy as a solution.

In this study, *S. equi* was the most frequently isolated respiratory pathogen (58.6%), supporting the findings of several other studies reporting high prevalence. Similar rates have been observed in Kansas 58%; (42), Egypt 54.8%; (43), and Sweden 50%; (44), However, lower *S. equi* prevalence has also been documented in UK 14.1%; (45), and Jammu 5%; (46). These differences may reflect variations in factors such as management practices, climate, or circulating *S. equi* strains.

*Klebsiella pneumoniae* was isolated in 31.03% of respiratory cases in this study. This prevalence is similar to that reported by Nehal et al. (47) in Egypt. However, other studies have reported lower *K. pneumoniae* prevalence, such as 3.3% in Austria (48) and 4.4% in Egypt (49). The rate of *Klebsiella* spp. isolation differs significantly from previous research, has been linked to factors like environmental conditions, climate, and sanitation managements.

The prevalence of *P. aeruginosa* in this study (31.03%) was higher than the 20.9% reported by Fonseca et al. (45) and the 3.8% by Nehal et al. (47). This higher prevalence may be related to *P. aeruginosa*’s ability to thrive in diverse environments, often acting as a nosocomial pathogen. *E. coli* was isolated in 24.13% of cases, a prevalence similar to that reported by Van Spijk et al. (50) in Switzerland. However, Fonseca et al. (45) reported a lower prevalence of 17.5%. The occurrence of *E. coli* in RTI is notable because it is considered a commensal organism on the equine mucosal membrane, as well as an opportunistic pathogen in respiratory diseases (50). We identified one case of mixed *K. pneumoniae* and *E. coli* infections in a horse with respiratory disorders. This finding is similar to that reported in Southern Brazil (51).

*Staphylococcus aureus* was isolated in 13.79% of cases. This is similar to the prevalence reported in India (52), but it is lower than 10.5 and 8.3% in Egypt (47) and (53). Higher rates were reported by Nwobi et al. (54) in Southeast Nigeria (23.9%) and in Central Ethiopia (16.7%) (55). Importantly, we also identified *S. aureus* coinfection with *P. aeruginosa* in two respiratory cases, consistent with the finding of Nehal et al. (47). The frequent human-horse interaction underscores the significance of *S. aureus* transmission between these species (52, 56).

In this study, the isolation rate of CoNS from respiratory infections was 3.44%. This is lower than the rates reported in previous studies: 17.3% (45), 14.28% (56), and 6.29% (52). In co5ntrast, Fernandes et al. (57) reported a much higher CoNS frequency of 36.6%. These differences in CoNS isolation rates may be due to variations in sample collection periods, horses’ clinical conditions, climatic changes, and sample size. Our finding of infrequent *S. pyogenes* occurrence in both single and mixed infections is consistent with the report by Borum (58) in urkey.

This study identified two *C. pseudotuberculosis* strains in the nostrils of horses (6.89%), consistent with the findings of Baraúna et al. (59), who detected the bacterium in the nasal sinuses or lungs of horses with internal abscesses. *C. pseudotuberculosis* causes respiratory disease in 40% of infected horses, resulting in pneumonia or pleuropneumonia (60).

Similar to our findings, previous studies have also identified *Acinetobacter* spp. from LRT and URT (5.5 and 12.2%, respectively) (45). Jokisalo et al. (61) isolated one MDR *A. baumanni* from the LRT, following treatment of primary pathogens such as *Rhodococcus equi* or *S. zooepidemicus.* This finding suggests that *Acinetobacter* spp. may emerge as secondary infections or indicate potential antibiotic resistance issues following treatment for other respiratory pathogens.

Polymicrobial respiratory infections were prevalent in this study, occurring at a rate of 62%. This finding is supported by previous research (45, 62). Mixed infections in equine are common and may develop to a severe case, a broad-spectrum antibiotic regimen is often recommended in this situation (45).

Guttural pouches, lined with respiratory mucosa, can become infected by any respiratory pathogen, leading to Guttural pouches empyema (GPE) (63). This study and others identified *S. equi, K. pneumoniae* and *S. aureus* as causative agents of GPE, with *S. equi* being the most prevalent (42, 64, 65).

Examination of various abscesses and wounds in Arabian horses identified several species, including *S. aureus*, *Corynebacterium* spp., *E. coli*, *P. aeruginosa*, *K. pneumoniae*, *S. equi*, and *S. pyogenes*. *S. aureus* was the most prevalent, occurring in 50% of pyogenic infection samples. *S. aureus* was the most frequently isolated bacterium, accounting for 41.66% of wound cases, this agree with Shuaib et al. (66) and higher than the 31% occurrence reported by Hussien Ahmed Mohammed (67). The presence of these microorganisms in equine wounds may be attributed to their pathogenic traits, such as enzyme and toxin production, which allows them to colonize and invade wounds more effectively than other bacteria (66).

*Escherichia coli* was isolated from 37.5% of wound cases, a finding consistent with Nadzir et al. (68) but higher than the prevalence reported by Shuaib et al. and Hussien Ahmed Mohammed (66, 67). *S. pyogenes* was isolated from 29.16% of cases. This prevalence is lower than the 41.2% reported by Hussien Ahmed Mohammed (67) but aligns with the findings of Shuaib et al. and Nadzir et al. (66, 68). This prevalence is likely due to *S. pyogenes* is a highly virulent and potentially fatal infection can arise from any open wound or other forms of non-penetrating trauma, like a contusion (66).

*Pseudomonas aeruginosa*, *K. pneumoniae*, and *S. equi* were present at lower prevalence (25%) in wound cases. These prevalence rates are exceeded previous studies (66, 68, 69), although the specific percentages varied. Further research is needed to understand the factors influencing the prevalence of these bacteria in equine wounds, including the potential roles of host immune responses and the wound environment.

*Corynebacterium pseudotuberculosis* was isolated from skin abscesses in 33.33% of cases. This finding is consistent with Akinniyi et al. (70), who isolated the bacterium from 10 years-old horse with forelimb abscess and pus discharge. Additionally, our study revealed a 37.5% prevalence rate of *Corynebacterium* spp. in wound cases, this prevalence is notably higher than the 5.9% reported by Hussien Ahmed Mohammed (67). While the precise route of infection remains uncertain, *C. pseudotuberculosis* is believed to enter the host through skin scratches (71).

High mortality rates associated with diarrhea in young horses significantly impact the equine industry and the livelihoods of breeders and farmers globally (72). This study found that Gram-negative bacteria, particularly *E. coli* (58.3%), were the most prevalent in foals with diarrhea. This prevalence was higher than that reported by reported by Haq et al. (72) (48.7%) but lower than the 81.7% reported by Adams (73). *P. mirabilis* and *K. pneumoniae* (33.3%) were the next most prevalent, exceeding the rates reported by Ata et al. (6) (25.72%) and Samir et al. (74) (20%). Co-infections, with *E. coli* and either *P. mirabilis* or *K. pneumoniae*, were more common than single infections, a finding consistent with Ata et al. (6). These co-infections may contribute to the severity or duration of diarrhea in foals.

Bacterial infections are a significant contributing factor to endometritis, infertility, and abortion in mares (8). In this study, *K. pneumoniae* was identified as the causative agent in 75% of late-term abortions (6–7 months). Co-infection with *S. equi* and *A. baumannii* was associated with abortion at 3.5 months, aligning with previous studies (8, 75). This study is the first to identify *A. baumannii* in vaginal swabs of pregnant mares, suggesting a potential role for *Acinetobacter* spp. in mare abortions. Further research is warranted to fully elucidate the mechanisms by which *A. baumannii* contributes to abortion in mares.

*Streptococcus pyogenes*, *E. coli*, *K. pneumoniae*, *Corynebacterium* spp. and *P. aeruginosa* were identified from eye swabs of infected horses. These findings corroborating previous studies that have also isolated these bacteria from equine eyes (14, 76). In addition, *E. coli* and *P. aeruginosa* were isolated from urine sample, a finding that aligns with previous reports of these bacteria in equine urinary tract infections (77).

All *S. equi* isolates in this study were resistant to both streptomycin and ceftiofur. This fonding is consistent with (45) who reported 100% streptomycin resistance in *S. equi* in the UK (78). observed different resistance pattern in Brazil, reporting 10% streptomycin resistance and 30% ceftiofur resistance in *S. equi* isolates. The widespread use of streptomycin in commercial penicillin formulations, may contribute to the high level of streptomycin resistance observed in this study. The unauthorized use of ceftiofur for treating *S. equi* infections may have contributed to the observed resistance to this antibiotic (45). In our study, penicillin, which is typically considered the preferred medication for treating non-pneumococcal streptococcal infections in horses, was found to be ineffective against 93.5% of *S. equi* and 100% of *S. pyogenes*. This contrasts with the findings of Veiga et al. (78), who observed resistance in 40% of *S. equi*, and Fonseca et al. (45) reported a 6.1% resistance rate in URT infections.

Borum (58) found complete resistance of *S. pyogenes* to penicillin. While tetracyclines are sometimes suggested as an alternative treatment for URT in horses (45). Our study found a high tetracycline resistance rate of 93.5%. This is higher than 66.7% resistance reported by Fonseca et al. (45) and the 20% reported by Veiga et al. (78).

Interestingly, *S. equi* exhibited high resistance to gentamycin (80%), exceeding 51.9% reported by Johns and Adams (69) and lower than 91.1% reported by Fonseca et al. (45). However, Veiga et al. (78) reported complete sensitivity to gentamicin. The widespread use of these antibiotics in the equine industry, likely due to their low cost and accessibility, may contribute to the observed levels of resistance.

Although enrofloxacin is not approved for use in horses, this study found a high resistance rate of 78%, suggesting its potential overuse or misuse. This resistance rate is substantially higher than the 10% reported by Veiga et al. (78) and the 18.5% reported by Johns and Adams (69).

We observed complete resistance of *S. equi* and *S. pyogenes* to amoxicillin-clavulanic acid, which is consistent with Nehal et al. (47) and contrasts with Borum (58), who reported complete sensitivity of *S. pyogenes* to this antibiotic. This high level of resistance to multiple ꞵ-lactam antibiotics, including penicillin, amoxicillin-clavulanic acid, and ampicillin, raises concerns about the efficacy of these commonly used drugs for treating *S. pyogenes* infections.

The high resistance of clinical *Streptococcus* spp. to third-generation cephalosporins (cefotaxime and ceftiofur) contradicts findings reported by Berwal et al. and Von Dollen et al. (79, 80), indicating that antibiotic activity was impacted by the tissue environment (80). The World Health Organization classifies ceftiofur as critically important for human medicine. Consequently, its application in veterinary practice should be discouraged (81). Furthermore, highly prevalence resistance to carbapenems was inconsistent with Abdel-Shafi et al. and Zhang et al. (82, 83), who reported MEM and IMI to be the most potent antibiotics against *S. pyogenes*.

This study observed an increase in MRSA and MRCoNS. Attia et al. (53) reported four MRSA strains in horses with respiratory illness in Egypt. Roudaud et al. (84) isolated three MRCoNS in Canada, and Othman et al. (85) reported 24 MRCoNS from horse’s nostrils in Libya. These studies, along with the present findings, highlight the global distribution of methicillin-resistant staphylococci in equine populations.

Fifth-generation cephalosporins, such as ceftaroline, are effective against MRSA (86), supporting our results that ceftaroline is the only *β*-lactam antibiotic effective against MRSA. Notably, the resistance rates of *S. aureus* and CoNS for clindamycin and quinupristin-dalfopristin were substantial in the present study, in contrast to Casagrande (87) who reported high sensitivity of both *S. aureus* and CoNS to these drugs. One VRSA isolate resistant to doxycycline, linezolid and clindamycin was identified, supporting Nehal et al. (47) in Egypt, who isolated eight VRSA stains resistant to these antibiotics. Most MRSA strains isolated from equines are sensitive to vancomycin, linezolid, and teicoplanin (77), supporting our findings regarding sensitivity to these drugs.

The high resistance rate of *S. aureus* isolates to erythromycin aligns with Roudaud et al. (84) in Canada (75%) and exceed the 66.7% reported by Begum et al. (88) in Bangladesh, the 50% in Turkey (58), and 16.9% in Nigeria (54).

However, CoNS exhibited complete resistance to erythromycin contrary to Borum, Casagrande, and Fonseca et al. (45, 58, 87). In contrast to earlier findings (87, 89), *S. aureus* exhibited high resistant to both rifampin and chloramphenicol in this study. Conversely, CoNS showed high sensitivity (75%) to these drugs, a finding consistent with Casagrande and Fonseca et al. (45, 87).

Resistance of *S. aureus* isolates to fusidic acid contrasts with Casagrande (87), where only 25% of isolates showed resistance, whereas CoNS isolates exhibited full resistance that exceeded Casagrande (87). *Staphylococci* can develop resistance to every clinically available antibiotic category, through spontaneous chromosomal mutations or by acquiring resistance determinants through horizontal transfer (86).

The rise of antibiotic resistance encompasses extended spectrum *β*-lactamase-producing *Enterobacteriaceae* (ESBL-E), which produce enzymes conferring resistance to cephalosporins, and aztreonam, and are inhibited by β-lactamase inhibitors (90). In addition, The resistance to β-lactam antibiotics is attributed to the inability of penicillins to effectively penetrate the bacterial outer membrane (77). Although, penicillins and cephalosporins are usually prescribed in equine medicine, high levels of resistance have been observed. In this study, the observed high resistance rate of *E. coli* and *K. pneumoniae* to ampicillin (96.9 and 100%, respectively) met the results of previous studies (50, 90), and exceeded another studies (6, 45, 47).

*Escherichia coli* and *K. pneumoniae* exhibited total resistance to Amoxicillin-clavulanic acid. This finding corroborating with several studies (50, 91), but differing from (92), who reported high sensitivity to this antibiotic.

Our results showed that *E. coli* isolates were completely resistant to all cephalosporins tested. This finding contrasts with the results reported by Shuaib et al. (66), who observed *E. coli* sensitivity to cefazolin, and de Lagarde et al. (91), who reported sensitivity to cefoxitin. Our findings also differ from the findings of previous studies (92, 93), who documented sensitivity of *E. coli* to cefazolin.

Similarly, *K. pneumoniae* displayed complete resistance to cephalosporins, contradicting Borum (58), who documented high sensitivity of *K. pneumoniae* to cefazolin. However, our results align with those of Loncaric et al. (48), who found all isolates resistant to ceftazidime. Importantly, we found that 66.67% of *E. coli* and 73.08% of *K. pneumoniae* isolates were resistant to ceftaroline, a broad-spectrum cephalosporin. Ceftaroline has shown varying efficacy against *Enterobacteriaceae* isolated from horses (94). The observed resistance to ceftaroline, along with the overall cephalosporin resistance, may be influenced by hospitalization and antibiotic use in equine settings.

Although carbapenem-resistant *Enterobacteriaceae* (CRE) have been reported from humans, pets, and farm animals, no equine cases have been reported in the United States (73). Similarly, the results regarding carbapenem sensitivity were favorable, corroborating previous research findings (48, 66, 90, 92).

Tetracycline and doxycycline resistance in *E. coli* and *K. pneumoniae* is a major issue due to the extensive use of tetracyclines in veterinary medicine. Several studies have documented the development of resistance in both species through various mechanisms (45, 50, 69, 95, 96). Our results confirm these previous observations. Despite this, Ata et al. (6) reported 77.7% sensitivity of *E. coli* to tetracycline. This difference in sensitivity might be explained by different tetracycline usage patterns in the studied regions.

Colistin, a last resort polymyxin antibiotic, is essential for treating severe MDR or XDR bacterial infections. The observed colistin resistance go hand in hand with Richter et al. (97).

Aminoglycosides effectively combat *Enterobacteriaceae*, with *E. coli* displaying high gentamicin sensitivity, consistent with previous studies (6, 66, 75, 98). However, contrasting studies reported gentamicin resistance among *E. coli* isolates ranging from 39.2 to 100% (50, 67, 69, 88). Moreover, *K. pneumoniae* showed 61.5% resistance met the results reported by van Spijk et al., Borum, abd Loncaric et al. (48, 50, 58).

Sulphonamide resistance, particularly to sulfamethoxazole-trimethoprim (SXT), frequently occurs in equine *E. coli* due to extensive antimicrobial use (77). Our results showed high prevalence of SXT resistance among *E.coli* (75.7%) and *K. pneumoniae* (76.9%) isolates, consistent with findings by other studies (50, 62, 90), and exceeding others (69, 91). In contrast, the high sensitivity of both isolates to SXT observed in previous studies (6, 45, 58).

*Proteus mirabilis* isolates demonstrated resistance to most antibiotics tested. This is consistent with previous findings (6, 48, 50, 90, 95), with some variations in susceptibility rates.

High resistance rates of *P. aeruginosa* isolates to cephalosporins, aztreonam, colistin, ticarcillin-clavulanic acid, and fosfomycin were observed, similar to the findings of Pottier et al. and Isgren et al. (95, 99), although the resistance rates varied. Our observed sensitivity to carbapenems, aminoglycosides, and fluoroquinolones was consistent with these studies. Aminoglycosides sensitivity was also similar to that reported by Fonseca et al. (45) but differed from the findings of van Spijk et al. (50).

*Acinetobacter baumannii* strains exhibited high resistance to most tested antibiotics. Our results are consistent with van Spijk et al. and Isgren et al. (50, 95) regarding high SXT resistance, but contrast with Jokisalo et al. (61). The observed susceptibility to gentamicin contrasts with Van Spijk et al. and Jokisalo et al. (50, 61). Furthermore, carbapenem resistance is a growing concern in *A. baumannii* isolates, often accompanied by co-resistance to all other antimicrobials. This observation is consistent with the findings of Van Der Kolk et al. (100).

Restriction analysis revealed that bacteriophages vB_Pae_LS225 and vB_Pae_LP125 were sensitive to the restriction enzymes *Hinf*I, *Hind*III, and *Hae*III, indicating their susceptibility to host bacterial restriction systems. Similarly, closely related phages vB_KpnS_FZ41, vB_KpnS_FZ10, and vB_KpnP_FZ12 exhibited similar restriction patterns (101). In agreement with these findings, Tan et al. (102) reported that all bacteriophages investigated in their study were digested by *Hae*III and *Hind*III. The observed variations in restriction digestion patterns among the different phage isolates reflect underlying genetic diversity. These results suggest that restriction enzyme analysis can serve as a rapid and cost-effective preliminary tool for differentiating bacteriophage isolates based on their genomic characteristics (102).

Plaque formation is the fundamental method for determining phage infectivity and host range. Moreover, the presence of a high number of tRNA genes in a phage genome is a common feature associated with broad host range. These tRNAs help the phage overcome differences in the host’s codon usage bias, compensating for host tRNA deficits, ensuring efficient translation of phage genes, subverting host defenses by counteracting tRNA-degrading enzymes (103).

In our study, both phages demonstrated lytic activity with host-specific plaque formation against five different bacterial species from equine infections. Similar findings have been reported by Tkhilaishvili et al. (104) who identified phage capable of infecting *S. aureus*/*P. aeruginosa* dual-species biofilms *in vitro*. Also, Yamaki et al. (105) described a broad-host-range phage that infected several bacterial species, including *E. coli* and *S. enterica*. Moreover, Manohar et al. (106) found that all used bacteriophages were effective against mixed bacterial population include: *E.coli*, *K. pneumoniae* and *Enterobacter* spp. These examples support the validity of our results and highlight the therapeutic potential of polyvalent phages. Our study lacks molecular evidence from the genomic analysis. While the presence of tRNAs is a strong indicator, further functional studies could provide more definitive evidence of their role in multi-host infection.

The observed antimicrobial resistance poses challenges in treating equine infections, emphasizing the importance of vigilant monitoring, prudent antibiotic use, and effective therapeutic strategies to protect horse health and curb the spread of resistant microorganisms. Given these challenges, bacteriophage therapy offers a promising alternative for combating AMR bacteria, particularly in wound treatment. Our use of a bacteriophage cocktail on wound surfaces showed promise. This approach is consistent with previous research by Sotnikova et al. (14), who successfully used a bacteriophage cocktail to treat ulcerative keratitis in equines. Furthermore, Marshall et al. (15) demonstrated the efficacy of phage cocktail in treating *Staphylococcal* superficial pyoderma in horses, achieving positive outcome. These data suggest that bacteriophage-based therapeutic agents hold significant potential for treating wound lesions. The use of convenience sampling for the study population, coupled with the lack of defined inclusion and exclusion criteria, means our findings may not be fully representative of all infected Arabian horses. This study’s limitations are the small sample size for treatment evaluation and lack the genomes sequences of phages.

## 5 Conclusion

In conclusion, our study reveals widespread antimicrobial resistance among Gram-negative and Gram-positive pathogens isolated from Arabian horses, raising concerns about zoonotic transmission and posing significant challenges to equine infection management. These findings underscore the critical need for vigilant antimicrobial resistance monitoring in equine settings to understand resistance mechanisms and develop effective strategies to mitigate its spread. Furthermore, our investigation suggests that bacteriophage-based therapies offer a promising alternative approach, particularly for treating wound infections in Arabian horses. Future research should focus on characterizing resistance mechanisms to refine these strategies and safeguard equine and human health.

## Data Availability

The original contributions presented in the study are included in the article/Supplementary material, further inquiries can be directed to the corresponding author/s.

## Footnotes

^1^ https://www.r-project.org/
