# Supplementary material for: Controlling drug-resistant bacteria in Arabian horses: bacteriophage cocktails for treating wound infections
Source: Front Vet Sci. 2025 Oct 15;12:1609955. doi: 10.3389/fvets.2025.1609955 (PMC12568036; doi:10.3389/fvets.2025.1609955)
Supplement: Supplementary file 1 [file Data_Sheet_1.docx]

Supplementary Material

**Table S1.** Clinical conditions and numbers of samples collected from farms in Egypt during August 2022 to August 2023.

| Clinical Conditions | Total Number | Locality | Age |
| --- | --- | --- | --- |
| Respiratory Manifestations | 29 | -Different stations in (Giza, Ismailia, and Sharkia).  -Private cases. | 1.5 months -14years. |
| Guttural pouch empyema | 5 | -Different stations in (Obour City and Giza). | Foals (2-8 months). |
| Pyogenic infections in different part  (Limbs, neck, teeth, buttocks, trunk, and hoof) | 18 | -Different stations in (Cairo, Giza)  -Private cases in Sharkia and Ismailia Government. | 9 months -18years. |
| Abortion at different stages of pregnancy (3.5 to 7months). | 4 | -Different stations in Cairo. | Adults (6 years-11years). |
| Diarrhoea | 12 | -Different stations (Cairo, Giza).  - Private cases in Sharkia Government. | Foals (10days -7months).  Adults (1-2 years) |
| Ocular disorders | 3 | -Different stations in (Cairo, Giza). | 7months -17years. |
| Wounds in different body parts (limbs, joints, and face) | 24 | -Different stations in (Cairo, Giza).  - Private cases in Sharkia Government. | 1month -21years. |
| Urinary tract infections (cystitis) | 1 | -Private case in Giza. | Adult 21 years |
| Navel ill | 2 | -Station in Cairo. | Foals (1- 2 months) |
| Wound between rectum and vagina (rectal tear) | 2 | -Station in Cairo  -Private case in Giza. | Adults (4years -7years) |
| Total numbers of samples | 100 | --- | --- |

**Table S2.** Target genes, oligonucleotide primers sequences, and expected amplicon sizes.

| Target gene | Primers sequences (5`–3`) | Amplicon size (bp^†^) | Tm^*^(^o^C) | Reference |
| --- | --- | --- | --- | --- |
| `*16S rRNA*  *K. pneumoniae* | F: ATT TGA AGA GGT TGC AAA CGA T | 130 | 57 | (1) |
|  | R: TTC ACT CTG AAG TTT TCT TGT GTT C |  |  |  |
| *16S rRNA*  *S. pyogenes* | F: CTA CTT GGA TCA AGA CGG GT | 419 | 37 | (2) |
|  | R: TTA GGG TTT CCA GTC CAT CC |  |  |  |
| *16S rRNA*  *E. coli* | F: CGCCTAATCCGCAACGTAAT | 140 | 50 | (3) |
|  | R: CGCAGCGTGATCCTGTTTAT |  |  |  |
| Sa0836  *S. aureus* | F: TCGAAATTAAATGTTGTCGTGTCTTC | 573 | 58 | (4) |
|  | R: TCATTTTTGACATGRAGAGAAACATC |  |  |  |
| *seeI*  *S. equi* | F: CGGATACGGTGATGTTAAAGA | 500 | 60 | (5) |
|  | R: TTCCTTCCTCAAAGCCAGA |  |  |  |
| *rpoB*  *corynebacterium* spp*.* | F: CGTATGAACATCGGCCAGGT | 446 | 58 | (6) |
|  | R: TCCATTTCGCCGAAGCGCTG |  |  |  |
| *narG*  *C. equi* | F: ACCCGTACTTGCACTCTTTC | 612 | 58 | (7) |
|  | R: AGTCAGTACTTCCGCAGGTC |  |  |  |
| *16S rRNA*  (*Pseudomonas* spp) | F: GACGGGTGAGTAATGCCTA | 618 | 54 | (8) |
|  | R: CACTGGTGTTCCTTCCTATA |  |  |  |
| *16S rRNA*  *P. aeruginosa* | F: GGGGGATCTTCGGACCTCA | 956 | 58 | (8) |
|  | R: TCCTTAGAGTGCCCACCCG |  |  |  |
| *16S rRNA*  *Acinetobacter baumanii* | F: CAGCTCGTGTCGTGAGATGT | 150 | 55 | (9) |
|  | R: CGTAAGGGCCATGATGACTT |  |  |  |
| *invA*  *Salmonella* spp. | F: GTGAAATTATCGCCACGTTCGGGCAA | 284 | 55 | (10) |
|  | R: TCATCGCACCGTCAAAGGAACC |  |  |  |
| *16S rRNA*  CoNS^§^ | F: GCGGTCCATCTATAAGTGA | 285 | 50 | (11) |
|  | R: GGGTGAGTAACACGTGGA |  |  |  |
| *ureR*  *Proteus* *mirabilis* | F: GGTGAGATTTGTATTAATGG | 225 | 58 | (12) |
|  | R: ATAATCTGGAAGATGACGAG |  |  |  |
| *lnuB*  *S. zooepidemicus* | F: ATGAAAGGGTGAAGAAATGTTA | 830 | 47 | (13) |
|  | R: CGTTACTCTCCTATTCACTAATGT |  |  |  |

**^†^**Bp: base pair, **^*^**Tm: temperature, **^§^**CoNS: coagulase negative *staphylococci.*

| **Parameter** | ***E. coli* & *S. aureus*** | ***Corynebacteria* spp. & *A. baumannii*** | ***Salmonella* spp. & *S. pyogenes*** | ***S. equi*, *Pseudomonas* spp., & *K. pneumoniae*** |
| --- | --- | --- | --- | --- |
| Initial Denaturation | 94°C for 15 min | 94°C for 15 min | 94°C for 15 min | 94°C for 15 min |
| Number of Cycles | 40 | 30 | 30 | 35 |
| Cycling Conditions | | | | |
| - Denaturation | 94°C for 15 s | 94°C for 15 s | 94°C for 15 s | 94°C for 15 s |
| - Annealing | 50°C for 30 s | 55°C for 30 s | 55°C for 30 s | 60°C for 30 s |
| - Extension | 72°C for 30 s | 72°C for 30 s | 72°C for 30 s | 72°C for 30 s |
| Final Extension | 72°C for 7 min | 72°C for 7 min | 72°C for 7 min | 72°C for 7 min |

**Table S3. Multiplex PCR amplification conditions for multi-organism panels**

**Table S4. Uniplex PCR amplification conditions for bacterial species**

| **Target species** | **Target gene** | **Initial Denaturation** | **Number of Cycles** | **Cycling Conditions (Denaturation, Annealing, Extension)** | **Final Extension** | **Reference** |
| --- | --- | --- | --- | --- | --- | --- |
| ***Corynebacterium ovis* biovar *equi*** | *narG* | 95°C for 4 min | 30 | 95°C for 30 s, 58°C for 30 s, 72°C for 1.5 min | - | (7) |
| ***Pseudomonas aeruginosa*** | 16S rRNA | 95°C for 2 min | 25 | 94°C for 20 s, 58°C for 20 s, 72°C for 40 s | 72°C for 1 min | (8) |
| **Coagulase-negative Staphylococci (CoNS)** | 16S rRNA | 95°C for 5 min | 30 | 95°C for 1 min, 55°C for 1 min, 72°C for 1 min | 72°C for 5 min | (11) |
| ***Salmonella enterica* spp.** | *invA* | 94°C for 4 min | 30 | 94°C for 40 s, 58°C for 1 min, 72°C for 20 s | 72°C for 10 min | (10) |
| ***Proteus mirabilis*** | *ureR* | 94°C for 4 min | 30 | 94°C for 40 s, 58°C for 1 min, 72°C for 20 s | 72°C for 10 min | (12) |

**References**

1. Liu Y, Liu C, Zheng W, Zhang X, Yu J, Gao Q, et al. Pcr Detection of *Klebsiella Pneumoniae* in Infant Formula Based on 16s–23s Internal Transcribed Spacer. *Int J Food Microbiol* (2008) 125(3):230-5.

2. Iwasaki M, Igarashi H, Hinuma Y, Yutsudo T. Cloning, Characterization and Overexpression of a *Streptococcus Pyogenes* Gene Encoding a New Type of Mitogenic Factor. *FEBS letters* (1993) 331(1-2):187-92.

3. El-Demerdash AS, Bakry NR, Aggour MG, Elmasry SS, Mowafy RE, Erfan A, et al. Bovine Mastitis in Egypt: Bacterial Etiology and Evaluation of Diagnostic Biomarkers. (2023).

4. Goto M, Takahashi H, Segawa Y, Hayashidani H, Takatori K, Hara-Kudo Y. Real-Time Pcr Method for Quantification of *Staphylococcus Aureus* in Milk. *J Food Prot* (2007) 70(1):90-6.

5. Båverud V, Johansson S, Aspan A. Real-Time Pcr for Detection and Differentiation of *Streptococcus Equi Subsp. Equi* and *Streptococcus Equi Subsp. Zooepidemicus*. *Vet Microbiol* (2007) 124(3-4):219-29.

6. Pacheco LG, Pena RR, Castro TL, Dorella FA, Bahia RC, Carminati R, et al. Multiplex Pcr Assay for Identification of *Corynebacterium Pseudotuberculosis* from Pure Cultures and for Rapid Detection of This Pathogen in Clinical Samples. *J Med Microbiol* (2007) 56(4):480-6.

7. Almeida S, Dorneles EM, Diniz C, Abreu V, Sousa C, Alves J, et al. Quadruplex Pcr Assay for Identification of *Corynebacterium Pseudotuberculosis* Differentiating Biovar Ovis and Equi. *BMC Vet Res* (2017) 13:1-8.

8. Spilker T, Coenye T, Vandamme P, LiPuma JJ. Pcr-Based Assay for Differentiation of *Pseudomonas Aeruginosa* from Other *Pseudomonas* Species Recovered from Cystic Fibrosis Patients. *Journal of clinical microbiology* (2004) 42(5):2074-9.

9. Higgins PG, Wisplinghoff H, Stefanik D, Seifert H. Selection of Topoisomerase Mutations and Overexpression of Adeb Mrna Transcripts During an Outbreak of *Acinetobacter Baumannii*. *J Antimicrob Chemother* (2004) 54(4):821-3. doi: 10.1093/jac/dkh427.

10. Oliveira SD, Rodenbusch CR, Cé MC, Rocha SLS, Canal CW. Evaluation of Selective and Non‐Selective Enrichment Pcr Procedures for *Salmonella* Detection. *Lett Appl Microbiol* (2003) 36(4):217-21. doi: 10.1046/j.1472-765X.2003.01294.x.

11. Edwards K, Kaufmann M, Saunders N. Rapid and Accurate Identification of Coagulase-Negative *Staphylococci* by Real-Time Pcr. *J Clin Microbiol* (2001) 39(9):3047-51.

12. Zhang W, Niu Z, Yin K, Liu P, Chen L. Quick Identification and Quantification of Proteus Mirabilis by Polymerase Chain Reaction (Pcr) Assays. *Ann Microbiol* (2013) 63:683-9.

13. Azpiroz MF, Burger N, Mazza M, Rodríguez G, Camou T, García Gabarrot G. Characterization of *Streptococcus Equi Subsp. Zooepidemicus* Isolates Containing Lnub Gene Responsible for the L Phenotype. *Plos one* (2023) 18(4):e0284869.

**Table S5.** Breakpoints used for interpreting antimicrobial susceptibility of each species

1. Breakpoints used for interpreting antimicrobial susceptibility of *Staphylococcus* spp. according to guidelines of Clinical and Laboratory Standards Institute (CLSI, 2024) and the European Committee on antimicrobial susceptibility testing (EUCAST) (14, 15).

| Antimicrobial agent | Category | Potency concentration (µg) | Abbreviation | Zone diameter (mm)* | | |
| --- | --- | --- | --- | --- | --- | --- |
|  |  |  |  | **S ^1^≥** | **I^2^** | **R^3^ ≤** |
| Gentamicin | Aminoglycosides | 10 | CN | 15 | 13-14 | 12 |
| Rifampin | Ansamycins | 5 | RA | 20 | 17-19 | 16 |
| Ceftaroline | Anti-MRSA cephalosporins | 30 | CPT | 25 | 20-24 | 19 |
| Cefoxitin | Anti-staphylococcal  β-lactams | 30 | FOX | 22 | - | 21 |
| Ciprofloxacin | Fluoroquinolones | 5 | CIP | 21 | 16-20 | 15 |
| Trimethoprim-sulphamethoxazole | Folate pathway inhibitors | 1.25/23.75 | SXT | 16 | 11-15 | 10 |
| Fusidic acid | Fucidanes | 10 | FA | 24 | - | 24 |
| Tigecycline | Glycylcyclines | 15 | TGC | 19 | - | 19 |
| Clindamycin | Lincosamides | 2 | DA | 21 | 15-20 | 14 |
| Erythromycin | Macrolides | 15 | E | 23 | 14-22 | 13 |
| Linezolid | Oxazolidinones | 30 | LNZ | 21 | - | 20 |
| Chloramphenicol | Phenicols | 30 | C | 18 | 13-17 | 12 |
| Fosfomycin | Phosphonic acids | 200 | FF | 34 | - | 33 |
| Quinupristindalfopristin | Streptogramins | 15 | SYN | 19 | 16-18 | 15 |
| Tetracycline  Doxycycline | Tetracyclines | 30 µg  30 µg | TE  DO | 19  16 | 15-18  13-15 | 14  12 |

1. Breakpoints used for interpreting antimicrobial susceptibility of *Enterobacteriaceae* according to guidelines of Clinical and Laboratory Standards Institute (CLSI, 2024) and the European Committee on antimicrobial susceptibility testing (EUCAST) (14, 15).

| Drug | Category | Potency concentration (µg) | Abbrev-  iation | Zone diameter (mm)* | | |
| --- | --- | --- | --- | --- | --- | --- |
|  |  |  |  | **S ^1^≥** | **I^2^** | **R^3^ ≤** |
| Gentamicin  Amikacin | Aminoglycosides | 10  30 | CN  AK | 15  17 | 13-14  15-16 | 12  14 |
| Ceftaroline (approved only for  *E. coli*, *K. pneumoniae*, *K. oxytoca*) | Anti-MRSA cephalosporins | 30 | CPT | 23 | 20-22 | 19 |
| Ticarcillin-clavulanic acid | Antipseudomonal penicillins + β-lactamase inhibitors | 75/10 | TIM | 20 | 15-19 | 14 |
| Imipenem  Meropenem | Carbapenems | 10  10 | IMI  MEM | 23  23 | 20-22  20-22 | 19  19 |
| Cefazolin | Non-extended spectrum  cephalosporins; 1^st^ and  2^nd^ generation cephalosporins | 30 | CZ | 23 | 20-22 | 19 |
| Ceftazidime  Cefepime | Extended spectrum  cephalosporins; 3rd and 4^th^ generation cephalosporins | 30  30 | CAZ  FEP | 21  25 | 18-20  19-24 | 17  18 |
| Cefoxitin | Cephamycins | 30 | FOX | 18 | 15-17 | 14 |
| Ciprofloxacin | Fluoroquinolones | 5 | CIP | 31 | 21-30 | 20 |
| Trimethoprim-sulphamethoxazole | Folate pathway inhibitors | 1.25/23.75 | SXT | 16 | 11-15 | 10 |
| Tigecycline | Glycylcyclines | 15 | TGC | 18 | - | 15 |
| Aztreonam | Monobactams | 30 | ATM | 21 | 18-20 | 17 |
| Ampicillin | Penicillins | 10 | AM | 17 | 14-16 | 13 |
| Amoxicillin-clavulanic acid | Penicillins + β-lactamase inhibitors | 20/10 | AMC | 18 | 14-17 | 13 |
| Chloramphenicol | Phenicols | 30 | C | 18 | 13-17 | 12 |
| Fosfomycin | Phosphonic acids | 200 | FF | 16 | 13-15 | 12 |
| Tetracycline  Doxycycline | Tetracyclines | 30  30 | TE  DO | 15  14 | 12-14  11-13 | 11  10 |

1. Breakpoints used for interpreting antimicrobial susceptibility of *Pseudomonas aeruginosa* according to guidelines of Clinical and Laboratory Standards Institute (CLSI, 2024) and the European Committee on antimicrobial susceptibility testing (EUCAST) (14, 15).

| Drug | Category | Potency concentration (µg) | Abbrev-  Iation | Zone diameter (mm)* | | |
| --- | --- | --- | --- | --- | --- | --- |
|  |  |  |  | **S ^1^ ≥** | **I^2^** | **R^3^ ≤** |
| Gentamicin  Amikacin | Aminoglycosides | 10  30 | CN  AK | 15  17 | 13-14  15-16 | 12  14 |
| Ticarcillin-clavulanic acid | Antipseudomonal penicillins  + β -lactamase inhibitors | 75/10 | TIM | 24 | 16-23 | 15 |
| Imipenem  Meropenem | Carbapenems | 10  10 | IMI  MEM | 19  19 | 16-18  16-18 | 15  15 |
| Ceftazidime  Cefepime | Extended spectrum  cephalosporins; 3rd and 4th  generation cephalosporins | 30  30 | CAZ  FEP | 18  18 | 15-17  15-17 | 14  14 |
| Ciprofloxacin | Fluoroquinolones | 5 | CIP | 25 | 19-24 | 18 |
| Aztreonam | Monobactams | 30 | ATM | 22 | 16-21 | 15 |
| Fosfomycin | Phosphonic acids | 200 | FF | 22 | 19-21 | 18 |

1. Breakpoints used for interpreting antimicrobial susceptibility of *Acinetobacter* spp. according to guidelines of Clinical and Laboratory Standards Institute (CLSI, 2024) and the European Committee on antimicrobial susceptibility testing (EUCAST) (14, 15).

| Drug | Category | Potency concentration (µg) | Abbrev-  Iation | Zone diameter (mm)* | | |
| --- | --- | --- | --- | --- | --- | --- |
|  |  |  |  | **S ^1^≥** | **I^2^** | **R^3^ ≤** |
| Gentamicin  Amikacin | Aminoglycosides | 10  30 | CN  AK | 15  17 | 13-14  15-16 | 12  14 |
| Ticarcillin-clavulanic acid | Antipseudomonal penicillins + β -lactamase inhibitors | 75/10 | TIM | 20 | 15-19 | 14 |
| Imipenem  Meropenem | Carbapenems | 10  10 | IMI  MEM | 22  18 | 19-21  15-17 | 18  14 |
| Ceftazidime  Cefepime | Extended spectrum cephalosporins; 3rd and 4^th^ generation cephalosporins | 30  30 | CAZ  FEP | 18  18 | 15-17  15-17 | 14  14 |
| Ciprofloxacin | Fluoroquinolones | 5 | CIP | 21 | 16-20 | 15 |
| Trimethoprim-sulphamethoxazole | Folate pathway inhibitors | 1.25/23.75 | SXT | 16 | 11-15 | 10 |
| Ampicillin-sulbactam | Penicillins + β-lactamase inhibitors | 10/10 | SAM | 15 | 12-14 | 11 |
| Tetracycline  Doxycycline | Tetracyclines | 30  30 | TE  DO | 15  13 | 12-14  10-12 | 11  9 |

1. Breakpoints used for interpreting antimicrobial susceptibility of *Streptococcus* spp. according to guidelines of Clinical and Laboratory Standards Institute (CLSI, 2024) and the European Committee on antimicrobial susceptibility testing (EUCAST) (14, 15).

| Drug | Category | Potency concentration (µg) | Abbrev-  iation | Zone diameter (mm)* | | |
| --- | --- | --- | --- | --- | --- | --- |
|  |  |  |  | **S ^1^≥** | **I^2^** | **R^3^ ≤** |
| Amoxicillin-clavulanate  Ampicillin  Penicillin | Penicillins  (alone or combined) | 20/10  30  10 IU | AMC  AM  P | 18  24  24 | 14-17  -  - | 13  -  - |
| Ceftiofur  Cefotaxime | Cephalosporins | 30  30 | EFT  CTX | 21  24 | 18-20  - | 17  - |
| Amikacin  Gentamicin  Kanamycin  Streptomycin | Aminoglycosides | 30  10  30  10 | AK  CN  K  S | 17  16  18  15 | 15-16  13-15  17  11-12 | 14  12  13  10 |
| Imipenem  Meropenem | Carbapenems | 10  10 | IMI  MEM | 26  26 | 23-25  23-25 | 22  22 |
| Enrofloxacin | Fluoroquinolones | 5 | ENR | 23 | 19-22 | 18 |
| Sulfamethoxazole-trimethoprim | Sulfonamides | 1.25/23.75 | SXT | 18 | - | 15 |
| Tetracycline | Tetracyclines | 30 | TE | 23 | 19-22 | 18 |

^1^S: sensitive,^2^I: intermediate.,^3^R: resistant.

*Zone diameter (mm) interpretation was based on zone diameter guidelines of Clinical and Laboratory Standards Institute (CLSI, 2024) and the European Committee on antimicrobial susceptibility testing (EUCAST) (14, 15).

14. CLSI. Performance Standards for Antimicrobial Susceptibility Testing. 34th ed. CLSI supplement M100. Clinical and Laboratory Standards Institute; 2024.

15. T﻿﻿he European Committee on Antimicrobial Susceptibility Testing. Breakpoint tables for interpretation of MICs and zone diameters. Version 11.0, 2021. (2021). Available at: <http://www.eucast.org>.

**Table S6.** Resistance rates of isolated Gram-negative bacteria against the tested antimicrobial agents and the multiple antibiotic resistance (MAR) index of the tested antimicrobials.

| AMA | No. of Gram-negative bacteria (%) (n=85) | | | | | *p*-value | MAR index^a^ |
| --- | --- | --- | --- | --- | --- | --- | --- |
|  | ***K. pneumoniae***  **(n= 26)** | ***E. coli***  **(n=33)** | ***P. aeruginosa***  **(n=20)** | ***Acinetobacter baunmannii***  **(n=2)** | ***Proteus mirabilis***  **(n=4)** |  |  |
| AM | 26 (100%) | 32 (96.97%) | - | - | 4 (100%) | 1 | 0.03 |
| AMC | 26 (100%) | 33 (100%) | - | - | - | NA | 0.03 |
| TIM | 26 (100%) | 31 (93.94%) | 20 (100%) | 2 (100%) | 4 (100%) | 0.429 | 0.04 |
| CZ | 26 (100%) | 33 (100%) | - | - | 4 (100%) | NA | 0.03 |
| FEP | 26 (100%) | 33 (100%) | 18 (90%) | 2 (100%) | 4 (100%) | 0.19 | 0.04 |
| CAZ | 26 (100%) | 33 (1000%) | 20 (100%) | 2 (100%) | 4 (100%) | NA | 0.05 |
| CPT | 19 (73.08%) | 22 (66.67%) | - | - | - | 0.405 | 0.02 |
| FOX | 26 (100%) | 30 (90.91%) | - | - | 4 (100%) | 0.384 | 0.03 |
| ATM | 25 (96.15%) | 16 (48.48%) | 18 (90%) | - | 4 (100%) | >0.0001^***^ | 0.03 |
| IMI | 0 | 0 | 3 (15%) | 2 (100%) | 1 (25%) | >0.0001^***^ | 0.003 |
| MEM | 0 | 0 | 1 (5%) | 0 | 0 | 0.306 | 0.001 |
| CT | 8 (30.77%) | 28 (84.85%) | 20 (100%) | 2 (100%) | 4 (100%) | >0.0001^***^ | 0.03 |
| CN | 16 (61.54%) | 7 (21.21%) | 8 (40%) | 0 | 4 (100%) | 0.001^**^ | 0.02 |
| AK | 5 (19.23%) | 2 (6.06%) | 5 (25%) | 1 (50%) | 0 | 0.152 | 0.007 |
| TE | 20 (76.92%) | 17 (51.5%) | - | 2 (100%) | 4 (100%) | 0.048* | 0.02 |
| DO | 20 (76.92%) | 17 (51.5%) | - | 2 (100%) | 4 (100%) | 0.048* | 0.02 |
| CIP | 14 (53.85%) | 13 (39.39%) | 6 (30%) | 0 | 4 (100%) | 0.035^*^ | 0.02 |
| SXT | 20 (76.92%) | 25 (75.76%) | - | 2 (100%) | 4 (100%) | 0.692 | 0.03 |
| C | 15 (57.69%) | 14 (42.42%) | - | - | 4 (100%) | 0.08 | 0.02 |
| FF | 26 (100%) | 33 (100%) | 20 (100%) | - | 4 (100%) | NA | 0.04 |
| TGC | 4 (15.38%) | 0 | - | - | 2 (50%) | >0.0001^***^ | 0.003 |
| SAM | - | - | - | 2 (100%) | - | NA | 0.001 |

AMA: antimicrobial agent, AM: ampicillin, AMC: amoxicillin-clavulanic acid, TIM: ticarcillin-clavulanic acid, CZ: cefazolin, FEP: cefepime, CAZ: ceftazidime, CPT: ceftaroline**,** FOX: cefoxitin, ATM: aztreonam, IMI: imipenem**,** MEM**:** meropenem**,** CT: colistin, CN: gentamycin, AK: amikacin**,** TE: Tetracycline**,** DO: Doxycycline**,** CIP: ciprofloxacin**.** SXT: trimethoprim-sulphamethoxazole**,** C: chloramphenicol**,** FF: Fosfomycin, TGC: tigecycline**,** SAM: ampicillin-sulbactam.

^a^MAR index for each antimicrobial agent = total number of resistance isolate to antimicrobial agent /total number of antimicrobials tested × total number of isolates (16). NA: non-applicable; **p* >0.05; ***p* >0.01; ****p* >0.0001.

16. Tambekar D, Dhanorkar D, Gulhane S, Khandelwal V, Dudhane M. Antibacterial Susceptibility of Some Urinary Tract Pathogens to Commonly Used Antibiotics. *Afr J Biotech,* (2006) 5(17).

**Table S7.** Effect of temperature on survival of phages

| **Temperature (****°C)** | **Phages (****Log PFU/mL)** | | ***p*-value** |
| --- | --- | --- | --- |
|  | **vB_Pae_LP125** | **vB_Pae_LS225** |  |
| Initial | 9.34±0.144^a^ | 8.18±0.104^a^ | 0.003^**^ |
| 30 | 9.23±0.318^a^ | 8.08±0.231^a^ | 0.043^*^ |
| 40 | 8.9±0.231^a^ | 7.85±0.364^a^ | 0.071 |
| 50 | 8.79±0.052^a^ | 7.48±0.173^ab^ | 0.002^**^ |
| 60 | 7.48±0.173^b^ | 6.85±0.289^b^ | 0.135 |
| 70 | 6.46±0.346^c^ | 0^c^ | >0.0001^***^ |
| 80 | 0^d^ | 0^c^ | NA |
| 90 | 0^d^ | 0^c^ | NA |
| 100 | 0^d^ | 0^c^ | NA |
| *p*-value | < 0.0001 | < 0.0001 |  |

PFU: plaque forming unit; NA: non-applicable. *, **, ***: indicates a significant difference between the effect of two phages at each point using independent sample T-test; * *p* < 0.05, ** *p* < 0.01, *** *p* < 0.001.

^a-d^ Means with different superscript letters within the same column indicate significant difference at *p* < 0.05 05 using one-way ANOVA, and Tukey’s post-hoc tests.

**Table S8.** Effect of time exposure to U.V. irradiation on survival of phage isolates

| **Time (minute)** | **Phages (Log PFU/mL)** | | ***p*-value** |
| --- | --- | --- | --- |
|  | **vB_Pae_LP125** | **vB_Pae_LS225** |  |
| Initial | 8.18±0.104^a^ | 9.34±0.173^a^ | 0.005^**^ |
| 20 | 8±0.289^a^ | 9.04±0.231^a^ | 0.048^*^ |
| 40 | 7.79±0.52^a^ | 8.92±0.462^a^ | 0.179 |
| 60 | 7.46±0.346^a^ | 8.71±0.346^a^ | 0.063 |
| 80 | 6.86±0.173^a^ | 7.3±0.231^b^ | 0.202 |
| 100 | 6.78±0.404^a^ | 6.97±0.289^b^ | 0.722 |
| 120 | 0^b^ | 0^c^ | NA |
| *p*-value | < 0.0001 | < 0.0001 |  |

PFU: plaque forming unit; NA: non-applicable. *, **, ***: indicates a significant difference between the effect of two phages at each point using independent sample T-test; * *p* < 0.05, ** *p* < 0.01, *** *p* < 0.001.

^a-c^ Means with different superscript letters within the same column indicate significant difference at *p* < 0.05 using one-way ANOVA, and Tukey’s post-hoc tests.

**Table S9.** Effect of pH values on different phage isolates

| **pH value** | **Phages (Log PFU/mL)** | | ***p*-value** |
| --- | --- | --- | --- |
|  | **vB_Pae_LP125** | **vB_Pae_LS225** |  |
| Initial | 8.18±0.104^a^ | 9.34±0.52^a^ | 0.094 |
| 2 | 0^d^ | 0^e^ | NA |
| 3 | 0^d^ | 4.92±0.346^d^ | >0.0001^***^ |
| 4 | 6.86±0.231^b^ | 7.3±0.173^bc^ | 0.202 |
| 5 | 7.11±0.063^b^ | 7.28±0.058^bc^ | 0.119 |
| 6 | 8.78±0.231^a^ | 8.72±0.231^a^ | 1 |
| 7 | 8.18±0.115^a^ | 9.34±0.289^a^ | 0.02^*^ |
| 8 | 7.28±0.173^b^ | 8.61±0.231^ab^ | 0.01^*^ |
| 9 | 6.86±0.173^b^ | 7.3±0.115^bc^ | 0.102 |
| 10 | 5.08±0.392^c^ | 6.75±0.519^c^ | 0.054 |
| 11 | 0^d^ | 0^e^ | NA |
| 12 | 0^d^ | 0^e^ | NA |
| 13 | 0^d^ | 0^e^ | NA |
| ***p*-value** | < 0.0001 | < 0.0001 |  |

PFU: plaque forming unit; NA: non-applicable. *, ***: indicates a significant difference between the effect of two phages at each point using independent sample T-test; * *p* < 0.05, *** *p* < 0.001.

^a-e^ Means with different superscript letters within the same column indicate significant difference at *p* < 0.05 05 using one-way ANOVA, and Tukey’s post-hoc tests.

**Table S10.** Adsorption rate of phages

| **Time (minute)** | **Phages (Log PFU/mL)** | | ***p*-value** |
| --- | --- | --- | --- |
|  | **vB_Pae_LP125** | **vB_Pae_LS225** |  |
| Initial | 8.18±0.115^a^ | 9.34±0.51^a^ | 0.095 |
| 1 | 6.95±0.404^ab^ | 7.9±0.404^b^ | 0.172 |
| 2 | 6.63±0.173^b^ | 7.11±0.16^b^ | 0.122 |
| 3 | 6.36±0.115^b^ | 7.15±0.087^b^ | 0.005^**^ |
| 4 | 6.32±0.462^b^ | 7.2±0.114^b^ | 0.138 |
| 5 | 5.78±0.289^b^ | 7.23±0.231^b^ | 0.017^*^ |
| ***p*-value** | 0.002 | 0.001 |  |

PFU: plaque forming unit. *, ***: indicates a significant difference between the effect of two phages at each point using independent sample T-test; * *p* < 0.05, *** *p* < 0.001.

^a-b^ Means with different superscript letters within the same column indicate significant difference at *p* < 0.05 05 using one-way ANOVA, and Tukey’s post-hoc tests.

**Table S11**. one-step growth of phages

| **Incubation period (minute)** | **Phages (Log PFU/mL)** | | ***p*-value** |
| --- | --- | --- | --- |
|  | **vB_Pae_LP125** | **vB_Pae_LS225** |  |
| Initial | 6.9 ±0.115^b^ | 5.74±0.138^b^ | 0.003** |
| 5 | 6.66±0.173^b^ | 6.4±0.115^b^ | 0.28 |
| 10 | 6.72±0.115^b^ | 6.11±0.116^b^ | 0.02^*^ |
| 15 | 7.61±0.535^ab^ | 7±0.289^b^ | 0.253 |
| 20 | 8±0.462^ab^ | 8.56±0.346^a^ | 0.387 |
| 25 | 8.52±0.173^a^ | 9.43±0.306^a^ | 0.061 |
| 30 | 8.86±0.404^a^ | 9.56±0.439^a^ | 0.306 |
| 35 | 8.91±0.346^a^ | 9.18±0.335^a^ | 0.605 |
| 40 | 8.18±0.104^ab^ | 9.43±0.421^a^ | 0.045^*^ |
| ***p*-value** | < 0.0001 | < 0.0001 |  |

PFU: plaque forming unit. *, ** Indicates a significant difference between the effect of two phages at each point using independent sample T-test; * *p* < 0.05, ** *p* < 0.01.

^a-b^ Means with different superscript letters within the same column indicate significant difference at *p* < 0.05 05 using one-way ANOVA, and Tukey’s post-hoc tests.

**
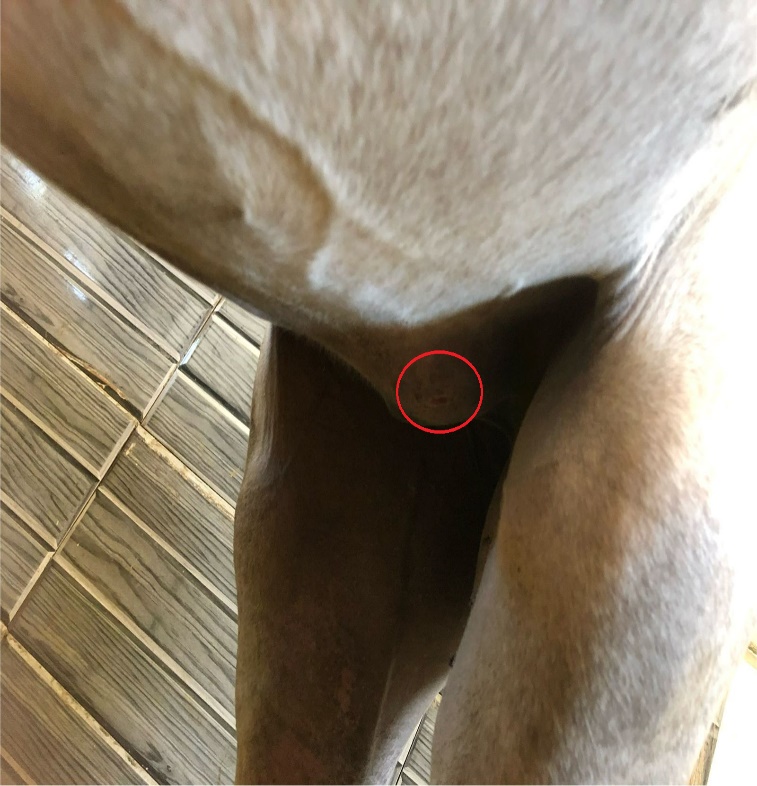

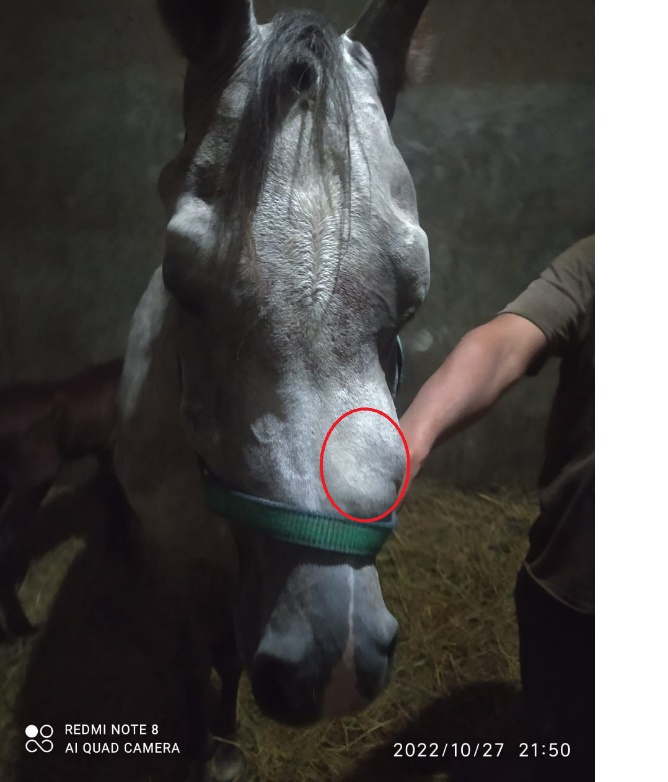
**
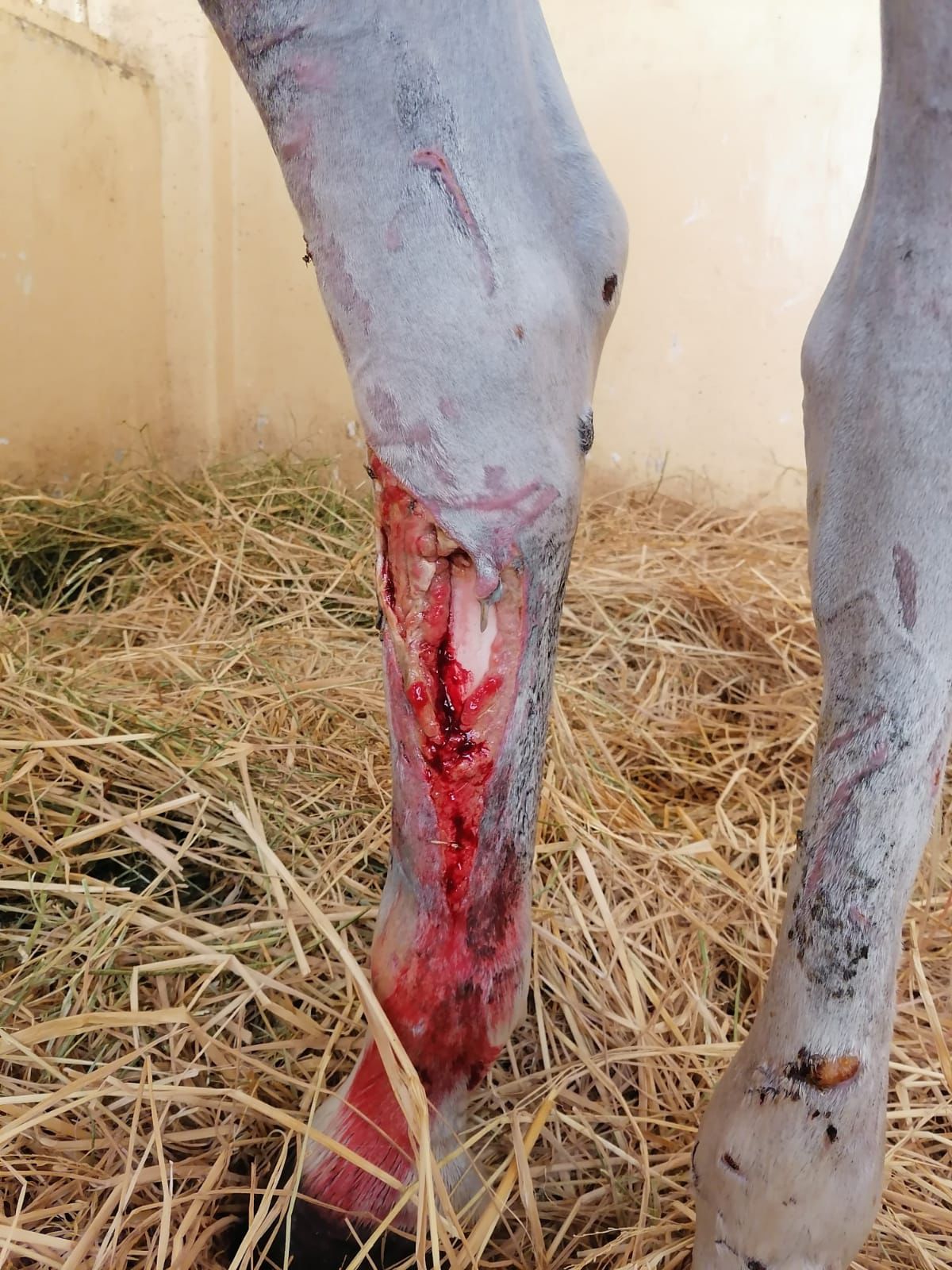

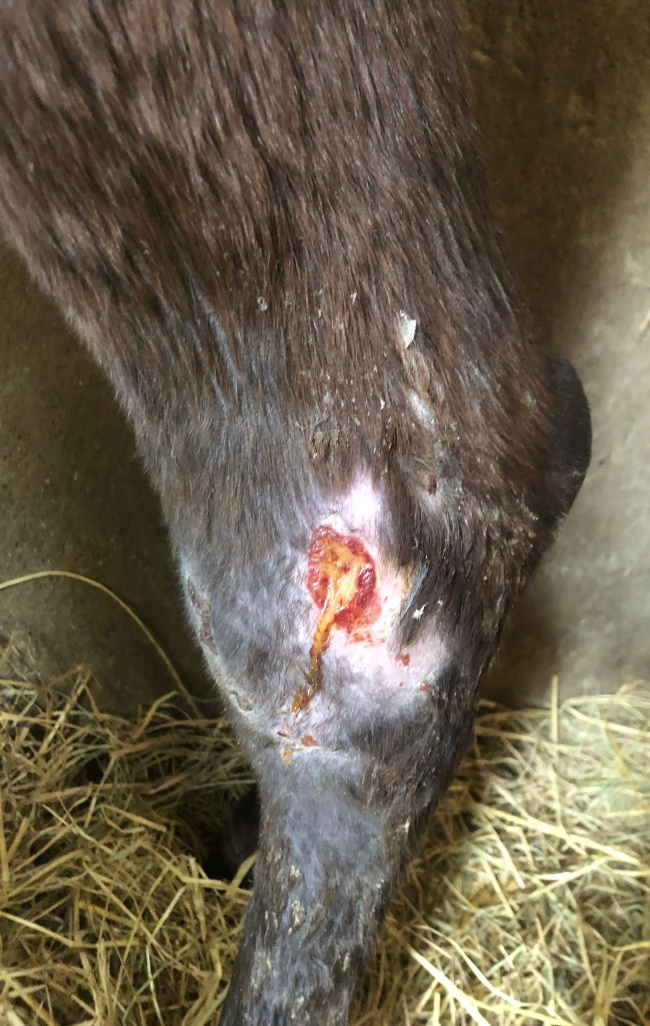


**D**

**C**

**A**

**B**

**Figure S1.** Arabian horses with infected wounds draining exudates (A) and pus (B) and abscesses (C and D).


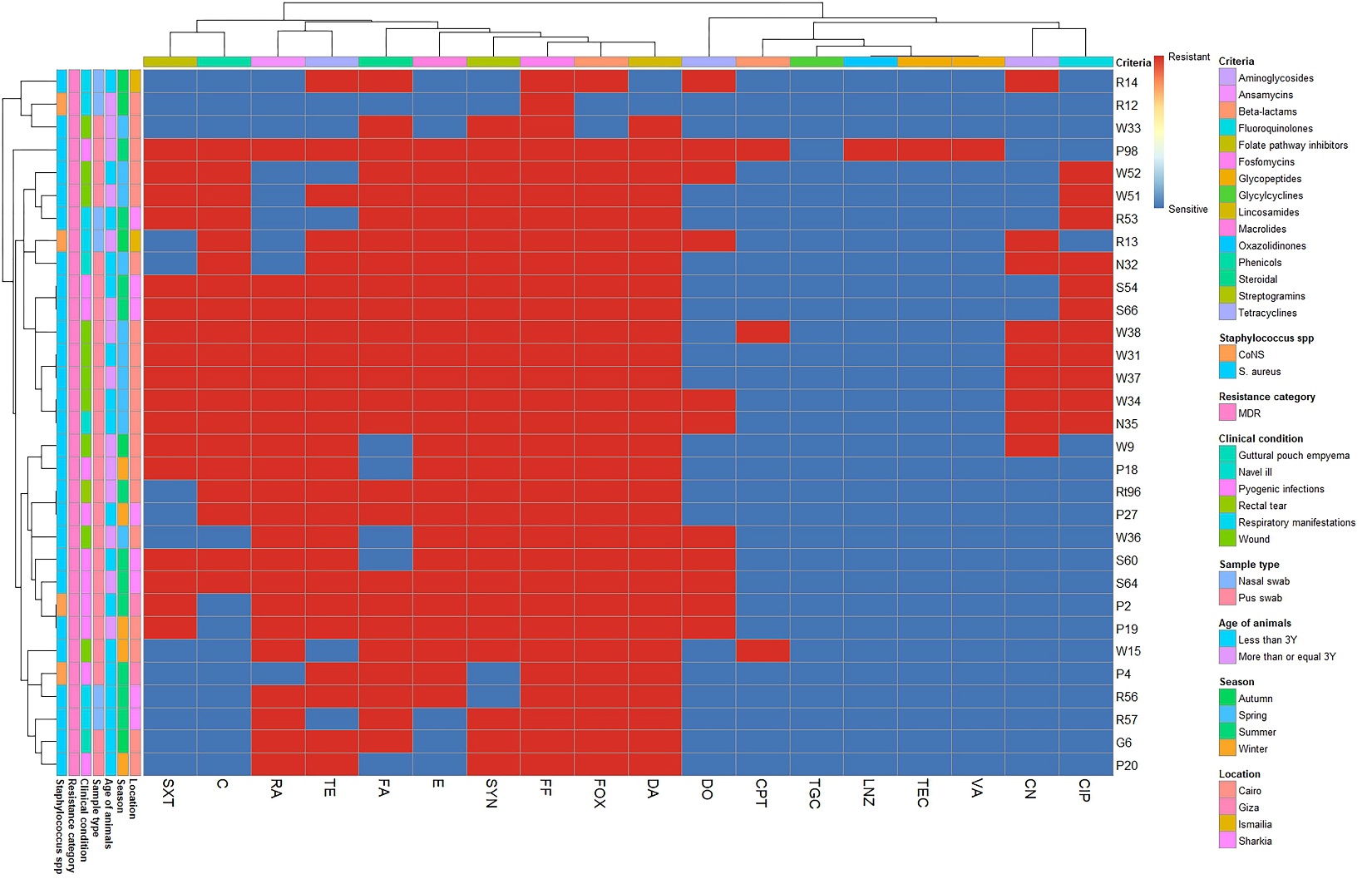


**Figure S2.** Overall distribution and clustering of *Staphylococcus* species isolates under study and the patterns of their antimicrobial resistance. Different *Staphylococcus* species, clinical condition, sample types, location, age of horse, season, antimicrobial classes, and resistance categories are shown for each isolate as colour codes. Red and blue colours refer to the resistance/sensitivity to an antimicrobial agent. The heatmap represents the hierarchical clustering of the isolates and the antimicrobials.

**
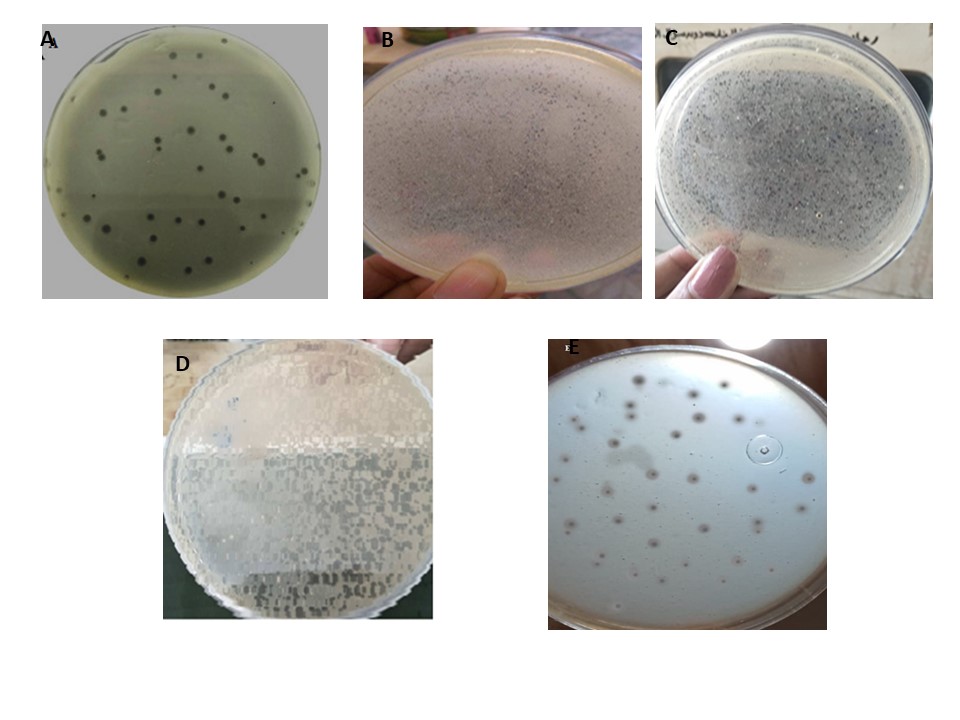
**

**Figure S3**. Plaque morphologies of bacteriophage vBPaeLS225 on nutrient agar against different bacterial hosts: (A) Medium, clear, circular plaques on *Pseudomonas aeruginosa*E8; (B) Small, turbid, irregular plaques on *Klebsiella pneumoniae* E11; (C) Small, turbid, irregular plaques on *Staphylococcus aureus* E18; (D) Large, clear, circular plaques on *Streptococcus pyogenes* E22; (E) Medium, clear, circular plaques on *Acinetobacter baumannii* E53.

**
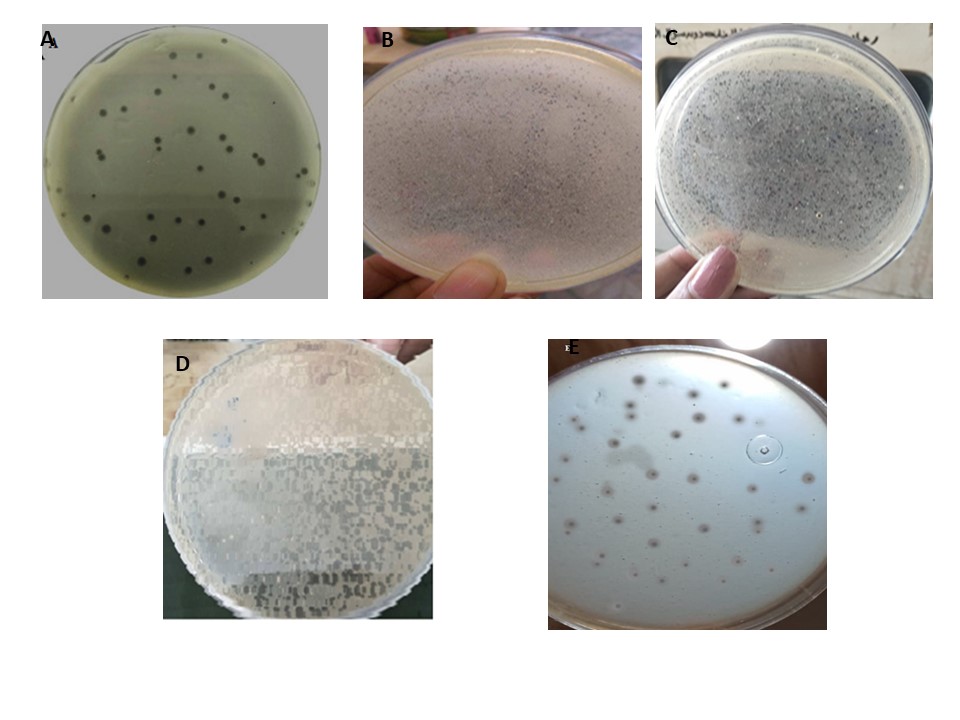
**

**Figure S4**. Plaque morphologies of bacteriophage vBPaeLP125 on nutrient agar against different bacterial hosts: (A) Small, turbid, irregular plaques on *Pseudomonas aeruginosa* E8; (B) Small, clear, circular plaques on *Klebsiella pneumoniae* E46. (C) Small, clear, circular plaques on *Staphylococcus aureus* E18. (D) Medium, clear, circular plaques on *Streptococcus pyogenes* E22. (E) Large, turbid, irregular plaques on the respective host.

**
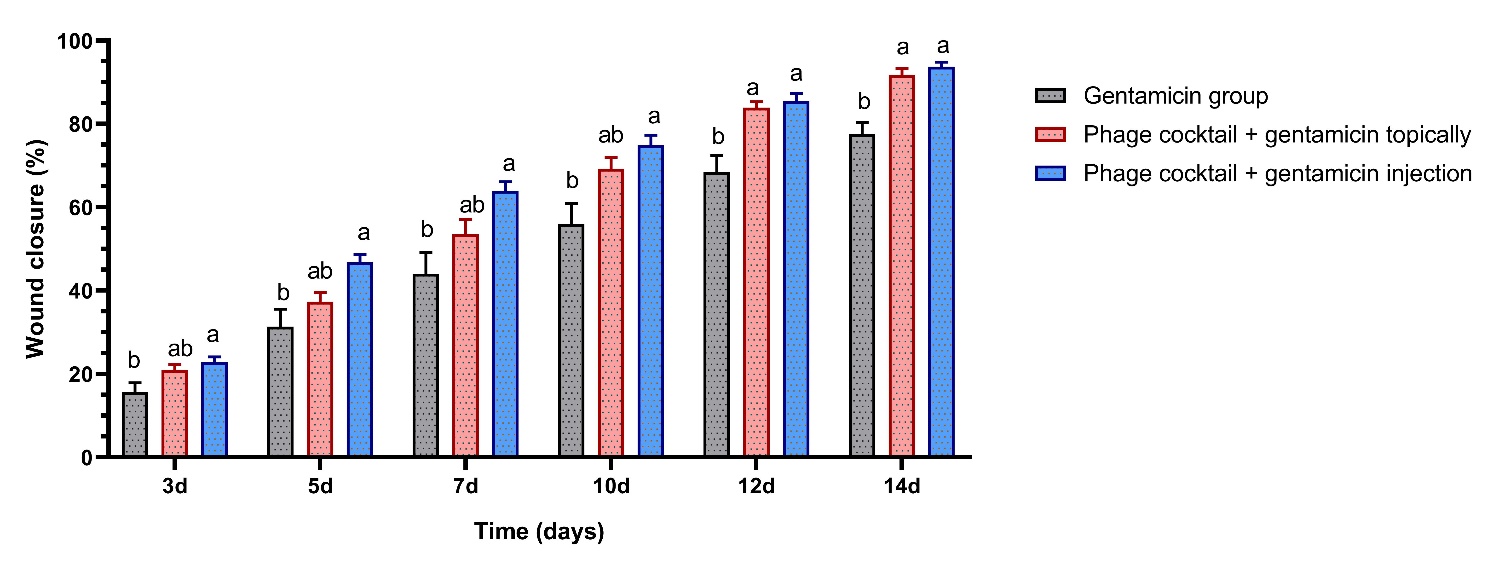
Figure S5**.The wound closure percentage in skin excisions during treatment in each group (n = 4) on days 3, 5, 7, 10, 12, and 14. Each value represents the mean ± standard error of the mean (SEM). ^a-b^ means with different superscript letters differ significantly at *p* < 0.05.
